# Europe’s land take and the loss of nature and cropland to artificial surfaces

**DOI:** 10.1038/s41467-026-71931-w

**Published:** 2026-04-13

**Authors:** Zander Samuel Venter, Bálint Czúcz, Anne Linn Kumano-Ensby, Léopold Salzenstein, Trond Simensen, Ruben Solvang, Mads Nyborg Støstad

**Affiliations:** 1 Norwegian Institute for Nature Research – NINA, Oslo, Norway; 2 Norwegian Broadcasting Corporation, Oslo, Norway; 3 Arena for Journalism in Europe, Amsterdam, Netherlands

**Keywords:** Environmental impact, Environmental economics, Environmental impact, Geography

## Abstract

The expansion of artificial surfaces, also known as land take, is a major contributor to habitat loss and biodiversity decline. Using high-resolution satellite data, we find that between 2018 and 2023 Europe lost 912 ± 74 km^2^ yr^−1^ of natural and semi-natural land cover and 588 ± 27 km^2^ yr^−1^ of cropland due to land take - nearly twice as large as previous estimates. Countries exhibiting the greatest land take per capita are Norway and Estonia, while Switzerland and Spain are most land efficient. The majority of land take (80%) occurs within modified ecosystems such as urban greenspace and semi-natural agricultural areas. Grasslands are disproportionately affected, with land take rates nearly five times that of other (semi-)natural ecosystems. The main land-use drivers of land take are residential (41%) and transport/logistics development (22%), and are therefore priority industries for targeting mitigation policies. Our methods provide a template for design-based monitoring of land take and call for an economic valuation of the ecosystem services lost due to land take.

## Abstract (web summary)

This paper analyzes high-resolution satellite data between 2018 and 2023, finding that Europe is losing nature and cropland to artificial land expansion at nearly twice the rate previously estimated.

## Introduction

The loss of natural habitat due to land-use change is the primary cause of biodiversity decline globally^1,2^, and a major contributor to carbon emissions and climate change^3^. To curb the trajectory of biodiversity loss, the Kunming-Montreal Global Biodiversity Framework (2022) has set an ambitious target to halt and reverse it by 2030^4^. However, our collective track record for adhering to international biodiversity targets is disappointing, with few countries being able to reverse trends in habitat loss^5^. This is especially concerning in Europe where only half of natural ecosystems remain^6^.

People have shaped European land systems for millennia, creating a continuum from undisturbed natural areas to semi-natural landscapes and heavily transformed urban or industrial zones characterized by artificial surfaces^7^. Artificialization, or in simpler words, land take is an important driver and component of habitat loss. In this process natural or semi-natural ecosystems (e.g., forests, grasslands, wetlands, etc.; henceforward *“natural and semi-natural land”*), and agroecosystems (actively managed arable and permanent crops; henceforward “*cropland”*) get destroyed for construction purposes and are entirely replaced with artificial ecosystems (i.e., built-up areas)^8,9^. While some of the area taken may be rehabilitated or recolonized later, portions of the eradicated vegetation will be permanently covered in non-permeable artificial materials (also known as *soil sealing*), resulting in a permanent loss of ecological functionality.

The European Commission has declared the ambitious goal for no net land take by 2050^10^. Robust monitoring of land take is crucial for tracking progress toward this target and for informing spatial planning and mitigation strategies. The failure to halt biodiversity losses can partly be attributed to the lack of clear and regularly updated data on land take, limiting civil society’s ability to hold governments accountable for meeting international targets^11,12^. State-of-the-art global maps of changes in urban land extent have been derived from Landsat satellite imagery with a pixel resolution of 30 m. More recently, a series of even higher resolution global land cover maps (10 m per pixel) have been produced based on Sentinel satellites, but are yet to be leveraged to quantify land take to date^13,14^. Many of the currently available European datasets, including CORINE Land Cover (CLC), the historically most long-standing solution to identify and map changes in land use and cover in Europe, do not allow the detection of fine-scale land-use and land cover changes (e.g. individual houses or small roads) contributing disproportionately to land take^15^. Other EU datasets, e.g. the CLC+ Backbone layers (CLC + BB), or the Copernicus Imperviousness Density (IMD) dataset leverage modern satellite imagery, combined with machine learning methods, which make a more comprehensive detection of land take possible.

An important limitation of satellite-derived land cover and land-use maps is that they cannot be directly used to measure land take. Such maps contain asymmetric errors, which means that simply adding up the areas covered by the map categories, a practice known as pixel counting, often leads to seriously biased area estimates^16^. Nevertheless, pixel counting is still a commonly used practice, partly even in official area statistics^17,18^. A robust alternative to simple pixel counting is design-based area estimation, which relies on a reference dataset covering a representative sample of the study area to yield unbiased area estimates along with statistically valid measures of their uncertainty^19^.

Another shortcoming of many available data sources is that they reveal little about the land-use drivers of land take^20^. Attributing the land-use drivers of loss and its spatial distribution help us answer the “why” and “where” questions which are vital for developing a mechanistic understanding of land take and for targeting mitigation actions accordingly^21^. Drivers can be classified as proximate and act locally, for example residential development, but often have distal causes that act regionally, for example national housing policies^20,22^. Linking land take to industry- and land-use-specific proximate drivers may be particularly helpful in Europe where EU-level trade and industry policy can have substantial impact on sustainable development targets.

In this study we combine automated land-take detection using modern AI-supported remote sensing products with a scalable approach for reference dataset creation to generate unbiased estimates of land take in Europe over the period 2018 to 2023. Specifically, we use the Dynamic World dataset by Google and the World Resources Institute^23^, to map land take over the study period for the region of the 39 collaborating countries (EEA-39) of the European Environment Agency (EEA). This region encompasses all the EU Member States, the United Kingdom, Iceland, Liechtenstein, Norway, Switzerland, Türkiye, and six Western Balkan countries (Albania, Bosnia and Herzegovina, Kosovo, Montenegro, North Macedonia, and Serbia). To correct for biases in the map and account for false positives and false negatives, we develop a web app supporting standardized identification of land take based on very high resolution aerial and satellite imagery. This app is deployed in collaboration with Arena for Journalism in Europe, an Europe-wide association of investigative journalists, who apply it on a stratified sample of 9,893 locations over all studied countries to produce unbiased estimates of land take (Supplementary Fig. 1). In addition, using the same app, the journalists also allocate land take to several types of proximate land-use drivers in a standardized and reproducible way.

## Results

### Land take and land-use efficiency

**Fig. 1:**
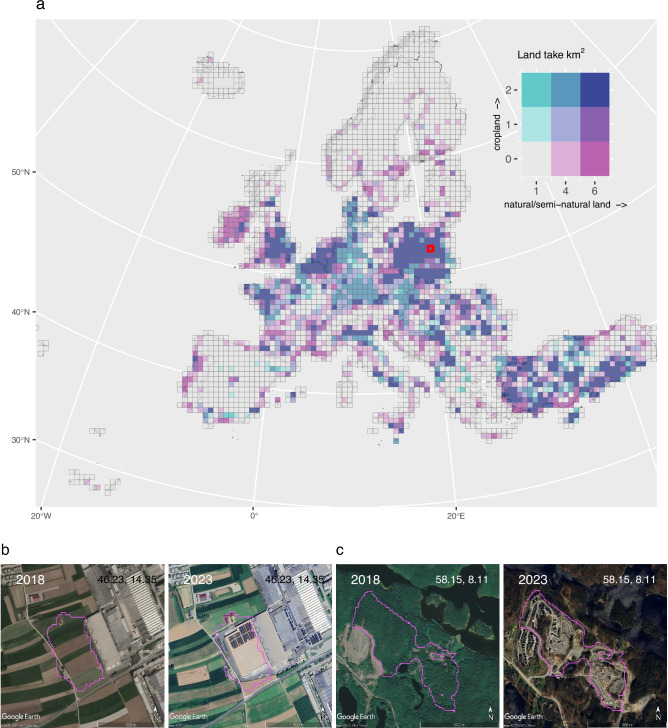
Map of land take within natural/semi-natural land and cropland. **a** Land take area (km^2^) for natural/semi-natural land and cropland aggregated to a 50 km grid. **b** Example of land take on cropland. **c** Example of land take on natural/semi-natural land. Aerial photographs: Google, @ 2025 Airbus. Eurostat GISCO geospatial data used for country boundaries licensed under a Creative Commons Attribution 4.0 International License (https://creativecommons.org/licenses/by/4.0/) © EuroGeographics for the administrative boundaries.

Using design-based area estimation to account for map biases, we estimate a total land take of 1548 ± 27 km^2^ yr^−1^ (±95% confidence interval) between 2018 and 2023 over the EEA-39 region (Supplementary Table 3). Artificial land cover increased by 3.75 ± 0.14% during this period (Supplementary Fig. 2b). When estimating land take of natural/semi-natural versus cropland land covers separately, we found that Europe lost 912 ± 74 km^2^ yr^−1^ of natural/semi-natural cover and 588 ± 27 km^2^ yr^−1^ of cropland due to artificial surface encroachment. When expressed relative to their baseline areas in 2018, Europe lost 0.09 ± 0.01% natural/semi-natural land cover and 0.23 ± 0.02% cropland cover (Supplementary Fig. 2c & d). The relative intensity of land take, and the balance between natural/semi-natural and cropland take, varied substantially over Europe (Fig. 1a). Land take regimes dominated by cropland loss were prevalent in Central-Northern Europe within countries like Germany and Denmark, which contain little natural/semi-natural cover to start with in 2018, and also have a high proportion of agricultural land. In contrast, land take regimes dominated by loss of natural/semi-natural cover were more dispersed over the whole of Europe, with hotspots in Ireland and Scandinavia, and coastal areas in Southern Europe. Areas exhibiting a strong coincidence of both cropland and natural/semi-natural land cover loss were located in the United Kingdom, Netherlands, Belgium, Poland and parts of Türkiye (Fig. 1a).

**Fig. 2:**
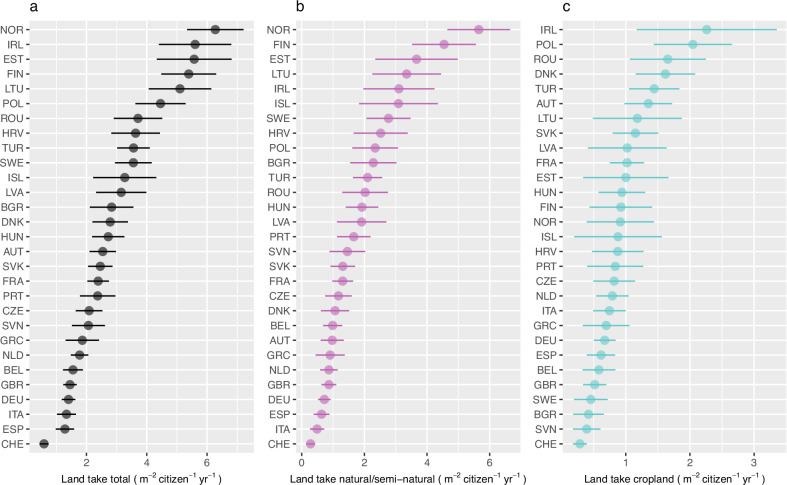
Area estimates of land take by country. **a** Land take expressed as an annual rate per capita, where population counts are from 2023. **b** Land take per capita on natural/semi-natural land. **c** Land take per capita on cropland (**c**). Unbiased area estimates are shown with points and 95% confidence intervals are shown with error bars. Confidence intervals are estimated from a sample size of *n* = 9893.

To enable comparison of land take rates across countries of different sizes, we expressed land take per capita using 2023 population figures, providing an indicator of land-use efficiency. The highest overall rates were observed in Norway (6.26 ± 0.94 m^2^ citizen^−1^ yr^−1^), Estonia (5.59 ± 1.23), and Ireland (5.46 ± 1.16), suggesting intensive land conversion relative to population size (Fig. 2a). In contrast, Switzerland (0.61 ± 0.15), Italy (1.34 ± 0.31), and Spain (1.29 ± 0.31) had the lowest per capita land take. When disaggregated by land cover source, Norway, Finland, and Estonia showed the highest land take on natural/semi-natural areas, while Switzerland, Italy, and Spain had the lowest (Fig. 2b). For land take on cropland (Fig. 2c), the highest rates were in Ireland, Poland and Romania, while the lowest were in Switzerland, Slovenia, and Sweden.

In addition to quantifying the per capita rate of land take, we also quantified land-use efficiency as marginal land consumption per unit change (i.e., growth or decline) in the population between 2018 and 2023 (Supplementary Fig. 3). Here, Finland consumed the most land compared to population growth over the same period. However, several countries exhibited land take despite population declines, thereby producing negative marginal consumption scores. Hungary topped the list with 250 m^2^ land take per citizen lost between 2018 and 2023 (Supplementary Fig. 3a). These patterns underscore not only spatial disparities in land take intensity but also varying levels of certainty across national estimates.

### Land take by ecosystem type and biome

To understand the environmental contexts in which land take is occurring, we quantified the proportional overlap between our map and a map of ecosystem types and another map of biomes (Table 1). We used the Corine Land Cover Accounting Layer (CLC AL) has been adopted for monitoring of ecosystem extent because it has a cross-walk to the ecosystem typology of Mapping and Assessment of Ecosystems and their Services (MAES)^6,24^. We used the RESOLVE Ecoregions map to define biomes^25^. While ecosystem types reflect functional and structural characteristics, such as land-use, vegetation cover, and management intensity, biomes capture broader climatic and ecological zones, offering a complementary lens on the natural context of land take.

**Table 1:** Input datasets used to define baseline land cover, ecosystem type and detect land take

Dataset	Period	Resolution	Purpose
Corine Land Cover backbone (CLC + BB)	2018	10 m	Defining baseline cropland and natural/semi-natural mask. Used for map strata of land take in natural/semi-natural land versus cropland.
Corine Land Cover Accounting Layer (CLC AL)	2018	100 m	Defining baseline ecosystem type
Dynamic World	2018–2023	10 m	Detecting land take
LUCAS	2018	point-based	Definition of artificial surface and cropland used in reference data collection and area estimation.

**Fig. 3:**
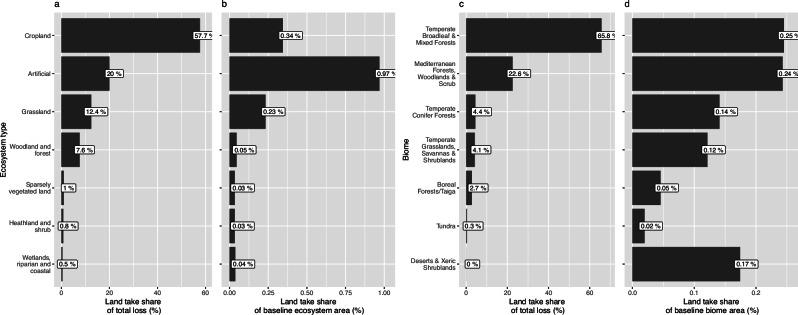
Proportional land take by ecosystem type and biome. **a** Percentage share of land take per ecosystem type. **b** Land take as a percentage of baseline ecosystem type area. **c** Percentage share of land take per biome. **d** Land take as a percentage of biome area (**d**).

The majority of land take (80%) is occurring within and around transformed ecosystems, such as urban green space and semi-natural agricultural areas (Fig. 3a). When expressed relative to their 2018 extent, artificial ecosystems experienced the most intense land take pressure (0.97%), followed by cropland ecosystems (0.34%; Fig. 3b), indicating that these ecosystems are disproportionately affected relative to their area coverage. Of the natural/semi-natural ecosystems, grasslands are experiencing rates of land take relative to their baseline area that are five times those of other natural/semi-natural ecosystems (0.23% versus 0.04–0.05%; Fig. 3b). Rarer ecosystem types, such as heathland, shrub, wetlands, coastal and riparian ecosystems (14% of Europe combined), are experiencing the least land take pressure.

The Temperate, Broadleaf and Mixed Forest biome contains two thirds of the land take observed in our map, although it covers 53% of Europe (Fig. 3c). When considering land take relative to total biome coverage, the Tundra and Boreal Forest/Taiga are the two biomes experiencing the least pressure from land take. In contrast, the two largest biomes (Temperate, Broadleaf and Mixed Forest, and Mediterranean Forests, Woodlands and Scrub) are experiencing the largest land take pressures.

### Proximate land-use drivers of land take

During the reference data collection for design-based area estimation, we collected information on land-use categories associated with each true-positive land take identified in the reference sample (Supplementary Table 5). We interpreted very high resolution satellite imagery, Google Street View and sometimes auxiliary information contained in Google Maps (e.g., points of interest or location names) to identify land uses. The underlying drivers of land take are complex and can range from European level policy mechanisms, via economic, cultural, and technological factors to individual landowner motivations. However, as a practical first step, we focus on land use as a proximate driver, since proximate drivers can be identified with remote sensing^20^.

**Fig. 4:**
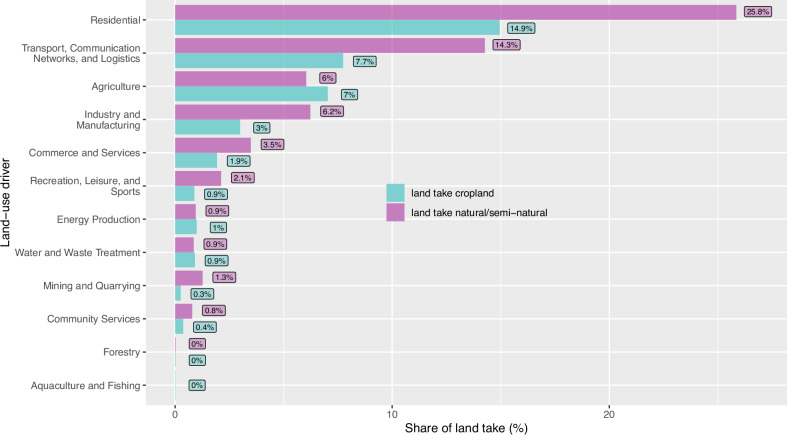
Land-use drivers of land take. Proportional contribution of land-use drivers to total land take.

We found that tertiary industry sectors including residential (including mixed urban areas and second homes) and transport/logistics development were responsible for the most land take (41 and 22%, respectively; Fig. 4). From the primary sectors, agriculture was the land use responsible for the most land take (13%), with forestry and mining constituting less than 1% of total land take (Fig. 4). Industry and manufacturing, a secondary land-use sector, followed agriculture with a 9.2% share of total land take.

### Map accuracy

The overall accuracy of our land take map, which included stable natural/semi-natural land and stable artificial/cropland classes, was 84.2% (± 1%). As expected, stable classes were mapped with much higher accuracy than the land take classes, which constituted less than 0.2% of the mapped area (Supplementary Table 3 and 4). User’s accuracy, measuring how often areas mapped as land take were correct based on the reference data, was 59% for total land take (combining natural/semi-natural land and cropland). Producer’s accuracy, indicating how often actual land take areas were correctly identified by the map, was 71%.

## Discussion

The EU’s Biodiversity Strategy for 2030 identifies land take as a key driver of biodiversity loss^26^, while the Soil Strategy for 2030 outlines a target of achieving no net land take by 2050^27^. The Soil Monitoring Law, adopted by the European Parliament on 23 October 2025, has less precise targets, but instead emphasizes a monitoring framework for the more visible aspects of land take: soil sealing and soil removal^28^. Beyond the focus on soil, the European Commission has introduced a Nature Restoration Law that aims to halt the decline of green urban areas by 2030, and increase their area by 5% by 2050^29^. However, our results show that these policy targets may be increasingly unrealistic. We found that Europe experienced an average of 1548 ± 27 km^2^ of land take annually between 2018 and 2023. In contrast, the EEA reports a gross land take of approximately 1000 km^2^ yr^−1^ between 2000 and 2018 (998 km^2^ yr^−1^ from the EEA statistical dashboard^17^ and 1160 km^2^ yr^−1^ in a recent scientific publication^6^). Although the aforementioned statistics are based on pixel counting, the EEA has done important work estimating the area of soil sealing using design-based methods, and report a rate of 413 km^2^ yr^−1^ between 2015 and 2018^18^. Soil sealing is one component of land take and therefore the estimates are not directly comparable with ours, however, our estimates are at least one and a half times larger than EEA land take estimates relying on coarse-resolution CLC Accounting Layers (Supplementary Fig. 4). Although we did not quantify net land take, the EEA estimates that 5% of gross land take is offset by artificial lands returning to cropland or natural/semi-natural land cover. If current trends continue, cumulative land take will overshoot the EU’s targets well before 2050.

Explaining the failed progress towards no-net land take targets requires understanding how land-use decisions are made. Land-use decisions are influenced by a complex interplay of cultural, economic, institutional, and environmental factors, with key drivers including population growth, urbanization, economic development and policy frameworks^20^. Notably, the countries with the highest land take per capita, are all sparsely populated countries. Norway, the country with the highest land take per capita, exemplifies how multiple factors contribute towards a contradiction between environmental commitments from the central government and excessive land take. While not directly assessed in our study, potential drivers of land use change in Norway include a low and dispersed population (18 people/km²), which increases per capita infrastructure needs. A strong economy, low income inequality, and cultural preferences for detached housing and second homes contribute to both urban expansion and rural sprawl^30^. A highly decentralized planning system^31^, along with strong bottom-up influence, also play a role, as 70% of Norwegian zoning plans are developer-initiated^32^.

Despite similar population growth rates to Norway, Switzerland exhibits the lowest rates of per capita land take. Switzerland’s approach to land-use is characterized by a collaborative effort between federal, cantonal, and municipal authorities, ensuring that land development aligns with national sustainability goals. Switzerland has shown relatively high compliance with strategic limits on the total area of building zones, as demonstrated in the canton of Zurich over a 20-year period^33^. However, attributing land-use efficiency solely to policy is likely premature. Factors, such as land scarcity, high land prices, and population density also strongly influence development patterns and the spatial footprint of urban growth^21,34^. Switzerland, for example, has among the highest population densities and topographical constraints in Europe, which may inherently limit expansive development. Further research is needed to quantify the relative contribution of planning frameworks versus structural drivers, such as land availability and settlement preferences.

We found that 80% of land take occurs in artificial and cropland ecosystems, as defined by CLC. Urban greenspaces, often misclassified as already “taken” in coarse-resolution land-use and cover maps, are essential for human well-being, biodiversity, and climate resilience^35,36^. Protecting these areas aligns with the Nature Restoration Law and is particularly urgent given the increasing densification of urban centers. Infilling and densification also happens on a smaller scale, and the trend termed “hidden urbanization”, whereby domestic gardens and residential areas are increasingly sealed with artificial and impervious surfaces can have substantial consequences for urban flooding^37–39^. Urban greenspaces also represent the most immediate nature exposure for most European citizens and provide critical ecosystem services, such as cooling, flood mitigation, and mental health support^36^. The loss of cropland is equally concerning. Soil sealing on fertile agricultural land reduces food production potential, undermining long-term food security—an issue that has gained political relevance amidst rising global uncertainty and supply chain instability^40^. Moreover, the EU Soil Strategy and the Soil Monitoring Law set clear objectives to reduce sealing rates, making cropland protection not just an environmental, but also an economic and geopolitical imperative.

Among (semi-)natural ecosystems, grasslands are under particularly high pressure, experiencing rates of land take five times greater than other natural/semi-natural classes. This is concerning given that some grassland types are biodiversity hotspots supporting a wide range of pollinators, soil organisms, and ground-nesting birds^41^. Grasslands also provide key ecosystem services including forage and fodder production, carbon storage, and water infiltration. Their apparent vulnerability to land take highlights the need for stronger protective measures, such as zoning restrictions and agri-environmental schemes targeted at maintaining semi-natural grasslands.

Our analysis further revealed that the most prominent land-use drivers of land take were residential (41%) and transport/logistics infrastructure (22%). Land efficient development within these sectors must therefore be central to mitigation strategies. Residential development often sprawls into peri-urban and rural areas, driven by housing demands and second-home ownership^42^. Addressing and limiting urban sprawl requires compact urban planning that balances densification patterns with conservation of urban greenspace^43^. Additional measures may include stricter zoning laws and incentives for brownfield redevelopment over greenfield expansion^44^. In several European countries, land reserves set aside for future development are often overestimated, leading to excessive zoning for land-use development and contributing to urban sprawl and land fragmentation^45^. For the transport sector, integration of infrastructure planning into broader sustainability frameworks and better use of existing corridors could reduce further encroachment into natural areas.

A major advance of our study lies in its methodological approach, which leverages cutting-edge technologies including modern remote sensing, artificial intelligence, reference data collection apps, and design-based area statistics, and combines them into a workflow producing unbiased area estimates for land take. This combination of methods is highly flexible: the final estimator pertains to the definition of land take applied in the reference dataset and can deviate from the definition used to create the map strata^19,46^. Therefore, it can be flexibly applied to any consistent and meaningful, but objectively interpretable definition of land take, or related processes, such as soil sealing, artificialization, etc. As comparing our results with similar other studies show, the details of the definition of land take (what is included and what is not) have a substantial influence on the final quantitative estimates, with permanently sealed surfaces only comprising up to ~30–40% of gross land take over Europe^18^. Our methodology offers relatively straightforward pathways for creating and comparing quantitative estimations for different operative definitions of land take in various geographical contexts. This could be leveraged for improving the clarity and standardization of key land take concepts in the future.

Furthermore, the whole methodology can be relatively rapidly dispatched for any area of interest, offering rapid development and update cycles. Traditional CLC, CLC + BB, and IMD all follow relatively long update cycles (6, 2, and 3 years, respectively), which hampers timely policy intervention. In contrast, our method leverages the Dynamic World dataset, which is updated continuously with Sentinel-2 acquisitions, and can be analyzed annually using design-based inference. Although design-based area estimation requires a probability sample, with the help of an efficient web app and a devoted pool of interpreters, the sampling burden becomes manageable and it can be adjusted depending on the desired geographic resolution (e.g., national vs. subnational estimates). In our study we relied on a group of forty knowledgeable, motivated, yet non-expert interpreters (investigative journalists), who each spent an average of 7.2 (± 7.4; standard deviation) hours processing samples. At an average rate of 1.7 (± 1.4) minutes per sampling point, completing the full set of nearly 10,000 sample units equaled about 40 days of work. Increasing the number of countries included or the number of strata (ie. map classes) for area estimation would require more samples for processing. Beyond that, increasing sample size would also increase the precision of the area estimates, although in our case, estimates were precise enough to detect inter-country differences in land take (Fig. 2).

Furthermore, unlike pixel-counting methods which may produce biased area estimates, our design-based estimator yields unbiased area measures along with valid confidence intervals. These uncertainty metrics add essential nuance for decision-makers and enhance the reliability of the results for reporting and planning. A further advantage of design-based methods is that although map errors may vary regionally depending on, for example, cloud and snow cover contamination of satellite imagery, the final area estimates per country will remain unbiased. Despite our efforts, such as seasonal filtering (Supplementary Fig. 5), some regional variation in land take detection accuracy may persist. However, our design-based sampling strategy, which stratifies by both country and map class, effectively corrects for such spatial biases in the final area estimates. As a result, any regional inconsistencies would primarily increase the uncertainty (wider confidence intervals) around the estimates, but would not systematically bias the estimates themselves.

However, a limitation of our study is that we estimated only gross land take and not net land take. Most notably, our methodology maps the “primary destruction” of (semi-)natural or agricultural ecosystems at sites of human intervention (construction), and does not consider that some of the “destroyed” area will be consciously or spontaneously revegetated in a few years’ time. Measuring this “primary loss” may be well justified from an ecological (biodiversity) perspective, but for other policy perspectives (e.g., consistency with long term land accounting) a more restrictive definition of land take can also be desirable. Nevertheless, mapping nature recovery on brownfields or actively restored areas remains a key gap and would require complementary analysis of vegetation regrowth and land use de-intensification over time. We also suggest that our dataset provides an opportunity for further studies on the underlying drivers of land take rates^21^, as well as displacement and leakage effects and land-use spillovers^47^.

The limited availability of accurate information on fine-scale land take covering Europe, and its land-use drivers, highlights a need to strengthen monitoring to track progress towards the EU’s ambition of achieving no net land take by 2050. Existing data products and validation workflows do not allow for timely monitoring of land take and neither can they efficiently connect them to proximate land-use drivers and economic sectors. Although land take covers a small portion of Europe’s territory annually, its impact on biodiversity and ecosystem services is disproportionately large due to its permanence and fragmentation effects. The cumulative nature of these small incursions creates a creeping loss of habitat connectivity and functional integrity. There is thus an urgent need for ecosystem accounts that not only track the spatial extent of land take, but also the ecosystem services and biodiversity values that are affected. Our results highlight the potential of modern satellite data and design-based estimation to provide this information and guide evidence-based mitigation across Europe.

## Methods

To map land take between 2018 and 2023, we used satellite remote sensing, machine learning and statistical sampling methods. Our analysis of land take is summarized in the flow diagram provided in Supplementary Fig. 1 and includes the following steps: (1) defining baseline land cover and ecosystem types in 2018; (2) mapping land take between 2018 and 2023; (3) defining map strata to estimate land take area per country and ecosystem type along with its land-use drivers; (4) collecting reference through a statistically sound sampling design and web application for image interpretation to validate the map; (5) applying design-based statistical techniques to evaluate the accuracy of the map and to produce unbiased estimates of land take area; (6) calculating land take rates relative to baseline land area and population statistics.

The study included an interpretative component; however, it did not involve the collection or processing of personal data, did not involve human participants as research subjects, and did not constitute health or medical research. As such, the project did not fall under regulations requiring formal ethics committee approval. The project was assessed in accordance with the Norwegian Institute for Nature Research’s institutional responsibility for research ethics and relevant national ethics guidelines prior to the commencement of the research and was deemed exempt from formal ethics committee approval. No additional permissions were required.

### Defining baseline land cover and ecosystem type

We chose 2018 as the start of our study period because it aligns with the production cycle of the EEA and Copernicus Land Monitoring Services where two important baseline maps are made available: (1) Corine Land Cover backbone (CLC + BB) 2018, and (2) Corine Land Cover Accounting Layer (CLC AL) 2018^48^. CLC + BB matches the spatial resolution of the land take map described in the following section (Table 1) and therefore formed the baseline map for identifying cropland and natural/semi-natural areas, where natural/semi-natural is anything but artificial and cropland (definitions in Tables 2 and 3).

**Table 2:** Definition of artificial/built-up surfaces across the land-use land cover datasets used in the study

Typology	Nomenclature	Code	Definition
Corine Land Cover	Artificial areas	111-142	Includes four subclases: (1) Urban fabric includes areas dominated by residential buildings and public infrastructure, along with related spaces, such as access roads and parking areas. (2) Industrial, commercial, and transport units cover zones used for manufacturing, business, finance, and major transport infrastructure like roads, railways, airports, and ports, as well as industrial-scale animal farming. (3) Mine, dump, and construction sites refer to lands primarily used for extraction, construction, or waste disposal activities. (4) Artificial non-agricultural vegetated areas are green spaces intentionally created for leisure or recreation, including urban parks and sports facilities.
Dynamic World	Built-up	6	Clusters of human-made structures or individual very large human-made structures. Contained industrial, commercial, and private building, and the associated parking lots. A mixture of residential buildings, streets, lawns, trees, isolated residential structures or buildings surrounded by vegetative land covers. Major road and rail networks outside of the predominant residential areas. Large homogeneous impervious surfaces, including parking structures, large office buildings, and residential housing developments containing clusters of cul-de-sacs.
LUCAS	Artificial land	A10-A30	Areas characterized by an artificial and often impervious cover of constructions and pavement. Includes roofed built-up areas and non-built-up area features, such as parking lots and yards. Includes non-built-up linear features, such as roads, and other artificial areas, such as bridges and viaducts, mobile homes, solar panels, power plants, electrical substations, pipelines, water sewage plants, open dump sites.

**Table 3:** Definition of cropland/agricultural areas across the land-use land cover datasets used in the study

Typology	Nomenclature	Code	Definition
Corine Land Cover	Agricultural areas	211-244	Includes four sub-classes: (1) Arable land consists of fields under rotation for annually harvested crops, including fallow lands and flooded crops like rice. (2) Permanent crops are areas used for long-term cultivation of fruit-bearing or woody plants, such as orchards, vineyards, and groves that are not rotated. (3) Pastures are lands used continuously for at least five years to grow forage, including natural or sown grasses and meadows, whether grazed or harvested. (4) Heterogeneous agricultural areas are complex mosaics where annual and permanent crops, meadows, and natural vegetation are intermixed or cultivated together, often within the same parcels or landscapes.
Dynamic World	Crops	4	Human planted/plotted cereals, grasses, and crops. Includes corn, wheat, soy, etc. Incluydes hay and fallow plots of structured land
LUCAS	Cropland	B10-B84	Areas where seasonal or perennial crops are planted and cultivated, including cereals, root crops, non-permanent industrial crops, dry pulses, vegetables, and flowers, fodder crops, fruit trees and other permanent crops. Includes temporary grasslands which are artificial pastures that may only be planted for one year. Includes permanent crops which are typically fruit trees, vineyards, olive groves.

In contrast to CLC + , CLC AL uses a coarser spatial resolution (Table 1) and takes a much broader spatial window into account when classifying a grid cell, with a minimum mapping unit of 25 ha (500 × 500 m). As a result, it captures landscape-level land cover types well and has been shown appropriate for defining the extent of ecosystem types in ecosystem accounting at the EU level^6^. We used a cross-walk between the CLC typology and the so-called MAES (Mapping and Assessment of Ecosystems and their Services) ecosystem typology^24^. The MAES typology is adopted in ecosystem accounting in the EU and, importantly, distinguishes grassland which is a class subsumed into agricultural and semi-natural areas in CLC (Supplementary Table 6).

### Mapping land take

While CLC AL has been updated every six years since 2000 and therefore allows for quantifying land take before 2018, its coarse spatial resolution means that fine-scale land take is unaccounted for. Although CLC+ is an improvement in spatial resolution from 100 m to 10 m, it has not been proven useful for monitoring land take at annual increments. Therefore, we used an alternative land-use land cover product, Dynamic World^23^ which is continuously updated with the Sentinel-2 image acquisitions (2015 to present). In the Dynamic World product, each Sentinel-2 satellite image is fed through a fully convolutional neural network (FCNN) trained on a global reference dataset of land-use land cover types. The FCNN performs multi-class segmentation of the satellite image producing probability scores for each 10 x 10 m pixel defining the probability of the pixel belonging to a given land-use land cover class. We processed the probability scores for the built-up class (Table 2) to isolate cases where a land-use land cover class changed from being not built-up to built-up between 2018 and 2023 (ie. land take). Specifically, we fitted a linear regression through the built-up area probability scores for each pixel, and then used a predefined threshold to identify positive linear trends that characterize land take. Because the Dynamic World model is run on all Sentinel-2 images during the year, there are many instances of snow cover or atmospheric contamination, such as cloud cover which artificially reduce the built-up probability scores for certain months of the year. Therefore, our algorithm contained two parameters which could be adjusted: (1) the linear trend threshold for defining built-up expansion for a pixel, and (2) the months within each year to filter Dynamic World images.

To identify the optimal linear trend threshold we produced an initial map of trends in built-up probability scores between 2018 and 2023 after filtering for images in June, July and August which approximate summer months over Europe and therefore reduce snow and cloud contamination. We used this map to produce a random calibration sample of 1000 pixels (10 x 10 m sample units) stratified over pixels with a positive linear trend in probability scores. The sample units were manually processed using visual interpretation of very high resolution aerial and satellite imagery to identify true or false cases of land take-, see section below on reference data collection for more details. These calibration data was used to identify the trend threshold which resulted in the most accurate land take detection. Accuracy was determined by constructing a confusion matrix (error matrix) with the land take classification at the given threshold, and the reference label. Producer’s accuracy measures the proportion of actual land take pixels correctly classified (i.e., the inverse of omission error), while user’s accuracy measures the proportion of pixels classified as land take that are correct (i.e., the inverse of commission error). The F1 score is the harmonic mean of producer’s and user’s accuracy, providing a balanced measure of classification performance that accounts for both omission and commission errors. The optimal linear trend threshold of 0.05 was identified (Supplementary Fig. 5).

The prevalence of snow and cloud cover, which can introduce noise in the built-up probability scores, vary substantially over Europe’s bioclimatic zones and geographies. Therefore we used a 100 × 100 km grid to identify locally-relevant months of the year for filtering Dynamic World built-up probability scores (Supplementary Fig. 5). Within each grid cell we stratified a random sample of 30 locations over areas mapped as artificial surface in CLC + 2018. We then extracted built-up probability score time series for all months in the year at the sample locations and isolated the three consecutive months in the year with highest median probability scores for built-up area across locations. To ensure that this resulted in a better land take map than simply including all months in the year, we quantified the accuracy using the 1000 sampling points stratified in the linear trend threshold optimization described above. This confirmed that filtering Dynamic World by locally-relevant month sets was most accurate (Supplementary Fig. 5).

The final land take map was produced with the optimal trend threshold and month subsets for all 39 countries in Europe mapped by CLC. However, the resulting map did not distinguish between land take on cropland versus natural/semi-natural land cover. We therefore overlaid our land take map with the baseline land cover map from CLC+ to identify pixels with cropland take and natural/semi-natural land take. Finally all water bodies including oceans were masked out from the analysis using CLC + . Therefore the scope of the analysis was restricted to terrestrial surfaces.

### Stratified sampling

The land take map produced could not be used directly for area estimation. Adding up pixel areas per mapped class, also known as pixel-counting, will be incorrect because of classification errors which are inevitable in all satellite-derived maps^16^. To quantify the accuracy of the land take map, and to produce area estimates that were not biased due to errors in the map, we followed good practices for design-based area estimation^19^. In statistical terms, a design-based area estimator is characterized as unbiased if it produces a parameter estimate such that the mean value taken over all possible samples is equal to the population parameter^49^. We implemented a stratified design and estimation approach^50^ to target the sampling of pixels exhibiting land take, which constitutes a very small fraction of the study area.

A goal of the analysis was to estimate land take per country and therefore we generated stratified sampling units (10 × 10 m pixels) per country using map strata including stable natural/semi-natural land, stable cropland and artificial surfaces, land take on natural/semi-natural land and cropland. The strata and sample allocations are defined in Supplementary Table 1. We started with the 30 countries in the European Economic Area plus Switzerland, Türkiye and the United Kingdom, but then excluded Lichetenstein, Luxembourg, Cyprus and Malta due to resource constraints in our project. These countries are smaller than 10,000 km^2^ and therefore would receive a disproportionate sampling effort to area ratio. We refer the reader to Supplementary Table 2 for the list of 29 countries included in the country-specific area estimation.

In addition to the stable and land take strata per country, we created buffer strata for each land take stratum. The buffer stratum was included to mitigate the anticipated impact of omissions of land take. Omission errors of small reference strata that occur in large mapped strata (e.g. true land take within area mapped as stable natural/semi-natural/cropland) have been reported and shown to introduce considerable uncertainty (i.e. low precision) in final area estimates^51^. The impacts of omission errors are mitigated if the large stratum is split into a substratum where omission errors are less likely to occur and a much smaller substratum where omission errors are more likely to occur. These buffer strata were not spatial buffers in the geometric sense, but were defined based on pixel-level trends in built-up probability. We defined the buffer stratum as pixels with intermediate trends in built-up probability scores of between 0.03 and 0.05. This buffer range was chosen based on visual inspection of the map where it was found that most land take omissions occurred within the 0.03 to 0.05 range. A thorough sensitivity analysis of varying buffer thresholds and widths would require a substantial amount of reference data collection, and empirical evidence for buffer selection guidelines are currently lacking^51,52^. Nevertheless, our inability to quantitatively prove the best buffer size (due to lack of stratified reference samples for each alternative buffer) does not invalidate the method. A poorly defined buffer will increase the standard error and widen the confidence interval because it reduces the likelihood of detecting omission errors, however it will not bias the area estimate itself^51,52^.

The total sample size for each stratum in each country was determined using the stratified variance estimator solved for n as advised by ref. ^51^, with a target standard error for overall accuracy of 0.02. Based on the formula, an optimal allocation is very close to an allocation proportional to the area of each stratum but with a minimum sample size of 40 units per stratum^51^ for the country-level sample. The total sample amounted to 9893 sampling units for reference data collection.

### Reference data collection

To facilitate the effective and standardized collection of high-quality reference data, we developed a labeling app built in Google Earth Engine. The app presented very high resolution reference imagery from Google Earth Online and ESRI Way Back to the interpreter, clearly highlighting the sampling unit in each of them. Where very high resolution imagery for 2018 or 2023 were unavailable, the interpreters used PlanetScope satellite imagery from Planet Labs, which provide 3 m resolution imagery that is sufficient to identify land take through visual interpretation. Interpreters were asked to follow a response design protocol which included identifying (1) whether the land cover in 2018 was cropland or natural/semi-natural; (2) whether land take took place between 2018 and 2023; and (3) the type of proximate land-use driver responsible for the land take.

To standardize the interpretation, the app also presented vital supporting information to standardize the identification of the focal process (land take), the two distinguished types of initial land cover (cropland vs. (semi-)natural land), and the different possible land-use drivers which had to be assigned to the sampling units with clearly detectable land take. The supporting information took the form of a cribsheet app which presented visual examples for the different land cover types and land-use drivers (terminal land use) within a 100 km proximity of the focal sampling unit. This was necessary because land cover classes in different European regions can look very different and therefore local context is important to aid interpretation. The visual examples were collected from sample locations in the 2022 LUCAS survey, which formed the basis for our definition of land take (Tables 2, 3 and Supplementary Table 5). We chose the LUCAS typology and survey data because it contains 337,854 locations on a systematic 2 km sampling grid visited and labeled in-situ in 2018 and are thus considered ground truths.

The app was then presented to a group of forty non-expert interpreters (investigative journalists), who also received a detailed initial training, and a continuous helpdesk support from the scientists participating in the study. Each sampling unit of the reference dataset was randomly assigned to an interpreter independent of their geographic location or home country. The following measures were taken to ensure the quality of the reference data: the interpreters were carefully trained to understand and identify the land-use land cover types, land take, and land-use drivers in the region; strata information was not made available during the collection of reference observations to ensure a blind collection; and the reference label was assigned one of four levels of confidence. Labels with the lowest confidence, or labels on which interpreters disagreed, were double-checked by experts at a later stage and modified.

### Unbiased area estimation

Following reference data collection, a stratified area estimator was constructed for estimation of map accuracy and area per mapped stratum, and confidence intervals were estimated using a stratified variance estimator^49^. At this stage the buffer strata were subsumed into the corresponding stable strata and the buffer areas were not estimated. The stratified estimator adjusts for the errors of omission and commission in the map and produces unbiased area estimates. In a statistical context, the term “estimator” used here refers to the formula by which estimates of population parameters are calculated from a reference sample. We performed stratified area estimation per country, and at the European level. To compare our land take results with those from EEA we needed to extrapolate from our sample of 29 countries to the 39 countries mapped by CLC. To do this we constructed an area estimator with our reference sample using area weights calculated from the 39 countries. Together, our sample of countries with reference data cover 96% of the land area mapped by CLC and was therefore deemed representative.

To attribute land take to direct land-use drivers, for each country we calculated the proportion of sample units verified as land take per land-use driver. For each land-use driver we calculated the sum of products between country-specific unbiased land take area and land-use driver proportion to estimate a total area for Europe.

Finally, according to recommended best practices^9^, we expressed land take as a rate per annum and expressed it relative to baseline land area or population size across countries. Specifically, we calculated (1) land take as a percentage of baseline terrestrial land area in 2018; (2) land take per capita based on population counts in 2023; (3) marginal land consumption defined as land take per capita change (ie. population growth or decline) between 2018 and 2023^53^. Country level population statistics were obtained from^54^. We also estimated land take area per ecosystem type and ecoregion as a proportion of the baseline class area.

### Validation and sensitivity analysis

The design-based area estimation produced unbiased measures of map accuracy per stratum per country (Supplementary Table 4). In theory, area estimates from a stratified estimator are robust to map inaccuracies, as they are derived from the reference sample rather than from mapped areas based on pixel counts^19^. However, map accuracy can influence the precision of the estimate, reflected in the width of the 95% confidence intervals.

In the initial sampling phase (1000 locations), we calibrated our algorithm to detect land take with optimal accuracy, identifying a linear trend threshold of 0.05 for built-up probability scores. Specifically, we identified a linear trend threshold for built-up probability scores of 0.05. To test the robustness of this threshold we performed a post-hoc sensitivity analysis using the full reference sample (*n* = 9893). We generated maps using linear trend thresholds ranging from 0.03 to 0.07, extracted area weights and post-stratified the original reference sample. Stehman et al.^46^ provide a post-stratified area estimator where the strata differ from the map classes. This estimator is appropriate for our sensitivity analysis given that the maps, generated with different trend thresholds, produce map classes for the reference sample. The post-stratified estimator produced unbiased map accuracy metrics per trend threshold which supported the choice of a 0.05 threshold. The 0.05 threshold produced a land take map with the highest F1 score and therefore the best balance between omission and commission error (Supplementary Fig. 6a). While the pixel counting area estimates steadily declined with increasing linear trend threshold the design-based estimates remained stable (Supplementary Fig. 6b).

### Reporting summary

Further information on research design is available in the Nature Portfolio Reporting Summary linked to this article.

## Supplementary information

Supplementary Information

Reporting Summary

Transparent Peer Review file

## Data Availability

The land take maps generated in this study have been deposited in the Zenodo database under accession code 10.5281/zenodo.17713896

## Code availability

The code to reproduce the analysis is located at GitHub, https://github.com/NINAnor/eu-dw-landtake

## References

[1] Díaz, S. et al. Pervasive human-driven decline of life on Earth points to the need for transformative change. *Science* **366**, eaax3100 (2019). 31831642 10.1126/science.aax3100

[2] Jaureguiberry, P. et al. The direct drivers of recent global anthropogenic biodiversity loss. *Sci. Adv.* **8**, eabm9982 (2022). 36351024 10.1126/sciadv.abm9982 PMC9645725

[3] Houghton, R. A. et al. Carbon emissions from land use and land-cover change. *Biogeosciences* **9**, 5125–5142 (2012).

[4] Leclère, D. et al. Bending the curve of terrestrial biodiversity needs an integrated strategy. *Nature* **585**, 551–556 (2020). 32908312 10.1038/s41586-020-2705-y

[5] Tittensor, D. P. et al. A mid-term analysis of progress toward international biodiversity targets. *Science* **346**, 241–244 (2014). 25278504 10.1126/science.1257484

[6] Ivits, E. et al. Twenty years of land accounts in Europe. *Land* **13**, 1350 (2024).

[7] Ellis, E. C. et al. People have shaped most of terrestrial nature for at least 12,000 years. *Proc. Natl. Acad. Sci. USA* **118**, e2023483118 (2021). 33875599 10.1073/pnas.2023483118 PMC8092386

[8] Decoville, A. & Feltgen, V. Clarifying the EU objective of no net land take: A necessity to avoid the cure being worse than the disease. *Land Use Policy* **131**, 106722 (2023).

[9] Marquard, E. et al. Land consumption and land take: enhancing conceptual clarity for evaluating spatial governance in the EU context. *Sustainability* **12**, 8269 (2020).

[10] European Commission. European Parliament and the Council Decision No 1386/2013/EU of the European Parliament and of the Council of 20 November 2013 on a General Union Environment Action Programme to 2020 ‘Living well, within the limits of our planet’. *Off. J. Eur. Union* 171–200 (European Commission, 2013).

[11] Andries, A., Morse, S., Murphy, R. J., Lynch, J. & Woolliams, E. R. Using data from Earth observation to support sustainable development indicators: an analysis of the literature and challenges for the future. *Sustainability* **14**, 1191 (2022).

[12] Pritchard, R., Sauls, L. A., Oldekop, J. A., Kiwango, W. A. & Brockington, D. Data justice and biodiversity conservation. *Conserv. Biol.* **36**, e13919 (2022). 35435288 10.1111/cobi.13919 PMC9796839

[13] Venter, Z. S., Barton, D. N., Chakraborty, T., Simensen, T. & Singh, G. Global 10 m land use land cover datasets: a comparison of dynamic world, world cover and Esri land cover. *Remote Sens.* **14**, 4101 (2022).

[14] Chakraborty, T. et al. Large disagreements in estimates of urban land across scales and their implications. *Nat. Commun.* **15**, 9165 (2024). 39448573 10.1038/s41467-024-52241-5 PMC11502887

[15] Haddad, N. M. et al. Habitat fragmentation and its lasting impact on Earth’s ecosystems. *Sci. Adv.* **1**, e1500052 (2015). 26601154 10.1126/sciadv.1500052 PMC4643828

[16] Venter, Z. S. et al. Uncertainty audit’for ecosystem accounting: Satellite-based ecosystem extent is biased without design-based area estimation and accuracy assessment. *Ecosyst. Serv.* **66**, 101599 (2024).

[17] European Environmental Agency. Land take and net land take. (EEA, 2019).

[18] Sannier, C., Ivits, E., Maucha, G., Maes, J. & Dijkstra, L. Harmonized pan-european time series for monitoring soil sealing. *Land* **13**, 1087 (2024).

[19] Olofsson, P. et al. Good practices for estimating area and assessing accuracy of land change. *Remote Sens. Environ.* **148**, 42–57 (2014).

[20] Plieninger, T. et al. The driving forces of landscape change in Europe: a systematic review of the evidence. *Land Use Policy* **57**, 204–214 (2016).

[21] Colsaet, A., Laurans, Y. & Levrel, H. What drives land take and urban land expansion? A systematic review. *Land Use Policy* **79**, 339–349 (2018).

[22] Lambin, E. F. & Meyfroidt, P. Global land use change, economic globalization, and the looming land scarcity. *Proc. Natl. Acad. Sci. USA* **108**, 3465–3472 (2011). 21321211 10.1073/pnas.1100480108 PMC3048112

[23] Brown, C. F. et al. Dynamic World, Near real-time global 10 m land use land cover mapping. *Sci. Data* **9**, 251 (2022).

[24] Maes, J. et al. Mapping and assessment of ecosystems and their services. * Anal. Framew. Ecosyst. Assess. Action* **5**, 1–58 (2013).

[25] Dinerstein, E. et al. An ecoregion-based approach to protecting half the terrestrial realm. *BioScience* **67**, 534–545 (2017). 28608869 10.1093/biosci/bix014 PMC5451287

[26] European Commission. *Communication from the Commission to the European Parliament, the Council, the European Economic and Social Committee and the Committee of the Regions. EU Biodiversity Strategy for 2030 - Bringing Nature Back into Our Lives*. (European Commission, 2020).

[27] European Commission. *Communication from the Commission to the European Parliament, the European Council, the Council, the European Economic and Social Committee and the Committee of the Regions ‘EU Soil Strategy for 2030 - Reaping the Benefits of Healthy Soils for People, Food, Nature and Climate’*. (European Commission, 2021).

[28] Panagos, P., Jones, A., Lugato, E. & Ballabio, C. A soil monitoring law for Europe. *Glob. Chall.* **9**, 2400336 (2025). 40071225 10.1002/gch2.202400336 PMC11891572

[29] Hering, D. et al. Securing success for the nature restoration law. *Science* **382**, 1248–1250 (2023). 38096279 10.1126/science.adk1658

[30] Overvåg, K., Xue, J., Steffansen, R. N. & Singsaas, M. Land use planning as an instrument for more environmentally sustainable second-home developments: hindrances and potentials. *Nor. Geografisk Tidsskr.-Nor. J. Geogr.* **78**, 210–221 (2024).

[31] Falleth, E. I. & Hovik, S. Local government and nature conservation in Norway: decentralisation as a strategy in environmental policy. *Local Environ.* **14**, 221–231 (2009).

[32] Stjernström, O., Junker, E. & Thorsen, H. W. The private in the public: the case of Norwegian private zoning plans. *Land Use Policy* **127**, 106585 (2023).

[33] Schmid, F. B., Kienast, F. & Hersperger, A. M. The compliance of land-use planning with strategic spatial planning – insights from Zurich, Switzerland. *Eur. Plan. Stud.* **29**, 1231–1250 (2021).

[34] Allan, A., Soltani, A., Abdi, M. H. & Zarei, M. Driving forces behind land use and land cover change: a systematic and bibliometric review. *Land* **11**, 1222 (2022).

[35] Jabbar, M., Yusoff, M. M. & Shafie, A. Assessing the role of urban green spaces for human well-being: a systematic review. *GeoJournal* **87**, 4405–4423 (2022). 34305268 10.1007/s10708-021-10474-7 PMC8290137

[36] Pinto, L. V., Inácio, M., Ferreira, C. S. S., Ferreira, A. D. & Pereira, P. Ecosystem services and well-being dimensions related to urban green spaces – A systematic review. *Sustain. Cities Soc.* **85**, 104072 (2022).

[37] Perring, M. P. et al. Advances in restoration ecology: rising to the challenges of the coming decades. *Ecosphere* **6**, 1–25 (2015).

[38] Verbeeck, K., Van Orshoven, J. & Hermy, M. Measuring extent, location and change of imperviousness in urban domestic gardens in collective housing projects. *Landsc. Urban Plan.* **100**, 57–66 (2011).

[39] Strohbach, M. W. et al. The “Hidden Urbanization”: trends of impervious surface in low-density housing developments and resulting impacts on the water balance. *Front. Environ. Sci*. **7**, 29 (2019).

[40] Basset, C. Soil security: The cornerstone of national security in an era of global disruptions. *Soil Security* **16**, 100154 (2024).

[41] Bengtsson, J. et al. Grasslands—more important for ecosystem services than you might think. *Ecosphere* **10**, e02582 (2019).

[42] Youngsteadt, E., Terando, A., Costanza, J. & Vukomanovic, J. Compact or sprawling cities: has the sparing-sharing framework yielded an ecological verdict? *Curr. Landsc. Ecol. Rep.* **8**, 11–22 (2023).

[43] Artmann, M., Inostroza, L. & Fan, P. Urban sprawl, compact urban development and green cities. How much do we know, how much do we agree? *Ecol. Indic.* **96**, 3–9 (2019).

[44] Morar, C., Berman, L., Unkart, S. & Erdal, S. Sustainable brownfields redevelopment in the European Union: an overview of policy and funding frameworks. *J. Environ. health* **84**, 24 (2021). 35350129 PMC8959022

[45] Kukulska-Kozieł, A. Buildable land overzoning. Have new planning regulations in Poland resolved the issue? *Land Use Policy* **124**, 106440 (2023).

[46] Stehman, S. V. Estimating area and map accuracy for stratified random sampling when the strata are different from the map classes. *Int. J. Remote Sens.* **35**, 4923–4939 (2014).

[47] Meyfroidt, P. et al. Focus on leakage and spillovers: informing land-use governance in a tele-coupled world. *Environ. Res. Lett.* **15**, 090202 (2020).

[48] European Environmental Agency. *Corine Land Cover Product User Manual*. https://land.copernicus.eu/en/technical-library/clc-product-usermanual/@@download/file (EEA, 2021).

[49] Cochran, W. G. *Sampling Techniques*. (Wiley, 1977).

[50] Olofsson, P., Foody, G. M., Stehman, S. V. & Woodcock, C. E. Making better use of accuracy data in land change studies: estimating accuracy and area and quantifying uncertainty using stratified estimation. *Remote Sens. Environ.* **129**, 122–131 (2013).

[51] Olofsson, P. et al. Mitigating the effects of omission errors on area and area change estimates. *Remote Sens. Environ.* **236**, 111492 (2020).

[52] Arévalo, P., Olofsson, P. & Woodcock, C. E. Continuous monitoring of land change activities and post-disturbance dynamics from Landsat time series: a test methodology for REDD+ reporting. *Remote Sens. Environ.* **238**, 111051 (2020).

[53] Schiavina, M., Melchiorri, M., Corbane, C., Freire, S. & Silva, F. B. e. Built-up areas are expanding faster than population growth: regional patterns and trajectories in Europe. *Journal of Land Use Science* https://www.tandfonline.com/doi/abs/10.1080/1747423X.2022.2055184 (2022).

[54] United Nations. World Population Prospects 2024. (United Nations, 2024).

