# Supplementary material for: Europe’s land take and the loss of nature and cropland to artificial surfaces
Source: Nat Commun. 2026 Apr 13;17:5122. doi: 10.1038/s41467-026-71931-w (PMC13247041; doi:10.1038/s41467-026-71931-w)
Supplement: Supplementary file 1 — Supplementary Information [file 41467_2026_71931_MOESM1_ESM.pdf]

# Europe’s land take: revealing loss of nature and cropland to artificial surfaces

## Supplementary Tables

*Table S1. Strata names and their description, strata weight (Wh [%]) based on the map of stable and change classes between 2018 and 2023, and number of sample units allocated (Nh).*

| Stratum | Description | Wh | Nh |
|---|---|---|---|
| stable natural/semi-natural | Natural/semi-natural land cover in 2018 and in 2023 | 74.1 | 3153 |
| stable artificial/cropland | Artificial or cropland in 2018 and in 2023 | 25.4 | 1358 |
| land take natural/semi-natural | Natural/semi-natural land cover in 2018 and artificial in 2023 | 0.15 | 1514 |
| land take natural/semi-natural buffer | Moderate probability for land take natural/semi-natural | 0.23 | 1177 |
| land take cropland | Cropland in 2018 and artificial in 2023 | 0.04 | 1490 |
| land take cropland buffer | Moderate probability for land take cropland | 0.09 | 1201 |

*Table S2. Strata allocation per country.*

| Country | stable natural/semi-natural | stable artificial/cropland | land take natural/semi-natural | land take cropland | land take natural/semi-natural buffer | land take cropland buffer |
|---|---|---|---|---|---|---|
| AUT | 105 | 41 | 41 | 42 | 45 | 41 |
| BEL | 75 | 47 | 44 | 40 | 43 | 43 |
| BGR | 88 | 43 | 47 | 40 | 44 | 40 |
| CHE | 107 | 39 | 40 | 40 | 42 | 41 |
| CZE | 80 | 47 | 45 | 41 | 47 | 41 |
| DEU | 92 | 67 | 59 | 42 | 54 | 44 |
| DNK | 56 | 67 | 45 | 40 | 41 | 40 |
| ESP | 150 | 53 | 62 | 41 | 67 | 44 |
| EST | 107 | 41 | 42 | 40 | 40 | 40 |
| FIN | 134 | 41 | 50 | 41 | 49 | 42 |
| FRA | 108 | 53 | 86 | 43 | 80 | 48 |
| GBR | 105 | 49 | 53 | 42 | 57 | 42 |
| GRC | 121 | 41 | 52 | 40 | 52 | 40 |
| HRV | 103 | 42 | 44 | 40 | 46 | 43 |

| | | | | | | |
|---|---|---|---|---|---|---|
| HUN | 69 | 64 | 53 | 41 | 43 | 40 |
| IRL | 118 | 42 | 45 | 40 | 47 | 39 |
| ISL | 139 | 39 | 41 | 40 | 42 | 40 |
| ITA | 120 | 48 | 57 | 42 | 80 | 42 |
| LTU | 85 | 41 | 41 | 40 | 42 | 41 |
| LVA | 106 | 40 | 43 | 40 | 42 | 42 |
| NLD | 78 | 46 | 45 | 40 | 45 | 41 |
| NOR | 156 | 41 | 44 | 41 | 42 | 39 |
| POL | 95 | 56 | 60 | 41 | 62 | 43 |
| PRT | 119 | 42 | 50 | 40 | 55 | 41 |
| ROU | 96 | 45 | 54 | 41 | 53 | 41 |
| SVK | 85 | 42 | 46 | 40 | 47 | 40 |
| SVN | 108 | 41 | 41 | 40 | 41 | 41 |
| SWE | 161 | 42 | 57 | 41 | 47 | 42 |
| TUR | 159 | 57 | 120 | 48 | 109 | 46 |

*Table S3. Confusion matrix of mapped strata at EU-level expressed in proportions, accuracies, unbiased area estimates and uncertainty of area estimates.*

| Reference class | | | | | | | |
|---|---|---|---|---|---|---|---|
| Map strata | stable natural/semi-natural | stable artificial/cropland | land take natural/semi-natural buffer | land take cropland buffer | land take natural/semi-natural | land take cropland | Total |
| stable natural/semi-natural | 0.63919 | 0.12646 | 0 | 0 | 0 | 0 | 0.7656 |
| stable artificial/cropland | 0.02602 | 0.20295 | 0 | 0 | 0 | 0 | 0.229 |
| land take natural/semi-natural buffer | 0.00137 | 0.00069 | 0 | 0 | 0.00026 | 0.00011 | 0.0024 |
| land take cropland buffer | 0.00017 | 0.0007 | 0 | 0 | 0.00005 | 0.00005 | 0.001 |
| land take natural/semi-natural | 0.00046 | 0.00025 | 0 | 0 | 0.00059 | 0.00025 | 0.0016 |
| land take cropland | 0.00004 | 0.00011 | 0 | 0 | 0.00007 | 0.00021 | 0.0004 |
| Total | 0.66725 | 0.33116 | 0 | 0 | 0.00097 | 0.000622217 | 1 |
| Area estimate (km2) | 630854 | 313094 | 0 | 0 | 913 | 588 | |
| 95% CI area estimate (km2) | 10139 | 10139 | 0 | 0 | 53 | 40 | |
| Margin of error | 0.02 | 0.03 | 0 | 0 | 0.06 | 0.07 | |
| User's accuracy | 0.835 | 0.886 | 0 | 0 | 0.378 | 0.499 | |
| Producer's accuracy | 0.958 | 0.613 | 0 | 0 | 0.608 | 0.339 | |

*Table S4. User's (UA) and Producer's (PA) accuracy per countries broken down into land take total, land take on natural/semi-natural land and land take on cropland.*

| Country | Land take total | | Land take natural/semi-natural | | Land take cropland | |
|---|---|---|---|---|---|---|
| | UA | PA | UA | PA | UA | PA |
| AUT | 0.72 (0.05) | 0.69 (0.05) | 0.32 (0.07) | 0.56 (0.1) | 0.81 (0.06) | 0.42 (0.06) |
| BEL | 0.61 (0.05) | 0.72 (0.07) | 0.47 (0.08) | 0.7 (0.08) | 0.45 (0.08) | 0.26 (0.07) |
| BGR | 0.46 (0.05) | 0.51 (0.07) | 0.34 (0.07) | 0.42 (0.08) | 0.38 (0.08) | 0.31 (0.1) |
| CHE | 0.62 (0.05) | 0.78 (0.08) | 0.28 (0.07) | 0.61 (0.13) | 0.58 (0.08) | 0.28 (0.06) |
| CZE | 0.56 (0.05) | 0.8 (0.06) | 0.32 (0.07) | 0.61 (0.09) | 0.39 (0.08) | 0.33 (0.08) |
| DEU | 0.7 (0.05) | 0.73 (0.05) | 0.44 (0.07) | 0.61 (0.08) | 0.66 (0.07) | 0.46 (0.06) |
| DNK | 0.53 (0.06) | 0.65 (0.06) | 0.26 (0.07) | 0.53 (0.11) | 0.6 (0.08) | 0.44 (0.07) |
| ESP | 0.49 (0.05) | 0.56 (0.06) | 0.24 (0.05) | 0.46 (0.1) | 0.49 (0.08) | 0.22 (0.05) |
| EST | 0.57 (0.06) | 0.7 (0.07) | 0.34 (0.07) | 0.59 (0.09) | 0.55 (0.08) | 0.27 (0.09) |
| FIN | 0.7 (0.05) | 0.74 (0.05) | 0.6 (0.07) | 0.68 (0.06) | 0.49 (0.08) | 0.27 (0.08) |
| FRA | 0.68 (0.04) | 0.76 (0.05) | 0.38 (0.05) | 0.61 (0.07) | 0.63 (0.07) | 0.32 (0.05) |
| GBR | 0.7 (0.05) | 0.81 (0.05) | 0.45 (0.07) | 0.74 (0.07) | 0.56 (0.08) | 0.31 (0.06) |
| GRC | 0.44 (0.05) | 0.62 (0.09) | 0.18 (0.05) | 0.44 (0.12) | 0.45 (0.08) | 0.24 (0.07) |
| HRV | 0.64 (0.05) | 0.72 (0.07) | 0.4 (0.08) | 0.55 (0.09) | 0.55 (0.08) | 0.35 (0.09) |
| HUN | 0.6 (0.05) | 0.73 (0.06) | 0.54 (0.07) | 0.76 (0.08) | 0.41 (0.08) | 0.26 (0.06) |
| IRL | 0.58 (0.05) | 0.75 (0.07) | 0.36 (0.07) | 0.8 (0.08) | 0.55 (0.08) | 0.1 (0.03) |
| ISL | 0.42 (0.06) | 0.83 (0.1) | 0.4 (0.08) | 0.84 (0.11) | 0.21 (0.07) | 0 (0) |
| ITA | 0.49 (0.05) | 0.63 (0.07) | 0.16 (0.05) | 0.45 (0.12) | 0.52 (0.08) | 0.24 (0.05) |
| LTU | 0.61 (0.06) | 0.79 (0.06) | 0.45 (0.08) | 0.78 (0.07) | 0.54 (0.08) | 0.32 (0.1) |
| LVA | 0.5 (0.06) | 0.67 (0.08) | 0.3 (0.07) | 0.63 (0.11) | 0.44 (0.08) | 0.09 (0.03) |
| NLD | 0.71 (0.05) | 0.74 (0.05) | 0.33 (0.07) | 0.57 (0.08) | 0.55 (0.08) | 0.25 (0.05) |
| NOR | 0.83 (0.04) | 0.62 (0.04) | 0.79 (0.06) | 0.61 (0.05) | 0.44 (0.08) | 0.17 (0.05) |
| POL | 0.55 (0.05) | 0.71 (0.05) | 0.34 (0.06) | 0.61 (0.08) | 0.55 (0.08) | 0.43 (0.07) |
| PRT | 0.49 (0.05) | 0.56 (0.07) | 0.41 (0.07) | 0.61 (0.09) | 0.25 (0.07) | 0.06 (0.02) |
| ROU | 0.53 (0.05) | 0.68 (0.06) | 0.29 (0.06) | 0.53 (0.09) | 0.38 (0.08) | 0.23 (0.06) |
| SVK | 0.69 (0.05) | 0.83 (0.05) | 0.46 (0.07) | 0.76 (0.07) | 0.56 (0.08) | 0.37 (0.06) |
| SVN | 0.55 (0.06) | 0.65 (0.08) | 0.37 (0.08) | 0.55 (0.1) | 0.51 (0.08) | 0.32 (0.1) |
| SWE | 0.66 (0.05) | 0.87 (0.05) | 0.52 (0.07) | 0.8 (0.06) | 0.56 (0.08) | 0.5 (0.15) |
| TUR | 0.53 (0.04) | 0.7 (0.04) | 0.36 (0.04) | 0.62 (0.06) | 0.4 (0.07) | 0.31 (0.05) |

*Table S5. LUCAS land use typology used to attribute land-use drivers of land take.*

| Sector | Code | land-use | Description |
|---|---|---|---|
| Primary | 111 | Agriculture | Land take associated with crop production, animal husbandry, and associated activities. Examples include crop cultivation greenhouses or tunnels, agricultural warehouses or storage facilities, infrastructure for raising livestock, and equipment storage areas or agricultural waste disposal areas. |
| | 120 | Forestry | Land take associated with growing timber, coppices, and forest products. Activities include planting, thinning, logging, and the storage of raw timber. Examples include forestry tracks and roads, timber processing plants, timber storage areas. |
| | 140 | Mining and Quarrying | Land take associated with the extraction of minerals, petroleum, natural gas, and other geological resources. Includes quarries for stone, sand, and gravel, as well as mining activities for coal, ores, and peat extraction. |
| Seconda ry | 210 | Energy Production | Land take associated with producing electricity, steam, and other forms of energy. Includes renewable energy facilities like wind and solar farms, fossil fuel plants, and hydropower stations, as well as biogas production sites. |
| | 221-228 | Industry and Manufacturing | Land take associated with the processing of raw materials and manufacturing products. Examples: food and beverage production facilities, textile factories, machinery manufacturing, chemical plants, wood-based product manufacturing, and printing and reproduction. Subcategories cover raw industries (e.g., smelting), heavy industries (e.g., vehicle production), and light industries (e.g., consumer goods). |
| Tertiary | 311-317 | Transport, Communication Networks, and Logistics | Land take associated with road, rail, air, and water transport, as well as pipelines and telecommunication networks. Includes parkng areas, airports, harbors, railway stations, and storage facilities for goods. |
| | 321-322 | Water and Waste Treatment | Land take associated with water supply and treatment facilities, sewer systems, and waste management plants. Examples include reservoirs, sewage treatment plants, and recycling centers. |
| | 340-342 | Commerce and Services | Land take associated with commercial activities like retail stores, offices, financial institutions, and professional services. Examples: shopping centers, banks, and office parks. |
| | 350 | Community Services | Land take associated with public institutions, such as schools, hospitals, police stations, and administrative offices. Examples: town halls, law courts, and public libraries. |
| | 361-363 | Recreation, Leisure, and Sports | Land take associated with parks, sports fields, amusement parks, and other areas for recreational or leisure activities. Examples: built-up surfaces in golf courses, swimming pools, and playgrounds. |

| | | | |
|---|---|---|---|
| | 370 | Residential | Land take associated with housing and residential purposes. Includes single-family homes, apartment complexes, recreational cabins, and mixed-use residential-commercial areas. |

*Table S6. Corine Land Cover ecosystem typology with crosswalk to MAES (Mapping and Assessment of Ecosystems and their Services) ecosystem typology.*

| CLC level 1 | CLC level 2 | CLC level 3 | MAES ecosystem type |
|---|---|---|---|
| Artificial surfaces | Urban fabric | 111 Continuous urban fabric | Urban |
| | | 112 Discontinuous urban fabric | |
| | Industrial, commercial and transport units | 121 Industrial or commercial units | |
| | | 122 Road and rail networks and associated land | |
| | | 123 Port areas | |
| | | 124 Airports | |
| | Mine, dump and construction sites | 131 Mineral extraction sites | |
| | | 132 Dump sites | |
| | | 133 Construction sites | |
| | Artificial, non-agricultural vegetated areas | 141 Green urban areas | |
| | | 142 Sport and leisure facilities | |
| Agricultural areas | Arable land | 211 Non-irrigated arable land | Cropland |
| | | 212 Permanently irrigated land | |
| | | 213 Rice fields | |
| | Permanent crops | 221 Vineyards | |
| | | 222 Fruit trees and berry plantations | |
| | | 223 Olive groves | |
| | Pastures | 231 Pastures | Grassland |
| | Heterogeneous agricultural areas | 241 Annual crops associated with permanent crops | Cropland |
| | | 242 Complex cultivation patterns | |
| | | 243 Land principally occupied by agriculture, with significant areas of natural vegetation | |
| | | 244 Agro-forestry areas | |

| | | | |
|---|---|---|---|
| Forest and semi natural areas | Forests | 311 Broad-leaved forest | Woodland and forest |
| | | 312 Coniferous forest | |
| | | 313 Mixed forest | |
| | Scrub and/or herbaceous vegetation associations | 321 Natural grasslands | Grassland |
| | | 322 Moors and heathland | Heathland and shrub |
| | | 323 Sclerophyllous vegetation | |
| | | 324 Transitional woodland-shrub | Woodland and forest |
| | Open spaces with little or no vegetation | 331 Beaches, dunes, sands | Sparsely vegetated land |
| | | 332 Bare rocks | |
| | | 333 Sparsely vegetated areas | |
| | | 334 Burnt areas | |
| | | 335 Glaciers and perpetual snow | |
| Wetlands | Inland wetlands | 411 Inland marshes | Wetlands |
| | | 412 Peat bogs | |
| | Maritime wetlands | 421 Salt marshes | Marine inlets and transitional waters |
| | | 422 Salines | |
| | | 423 Intertidal flats | |
| Water bodies | Inland waters | 511 Water courses | Rivers and lakes |
| | | 512 Water bodies | |
| | Marine waters | 521 Coastal lagoons | Marine inlets and transitional waters |
| | | 522 Estuaries | |
| | | 523 Sea and ocean | |

## Supplementary Figures

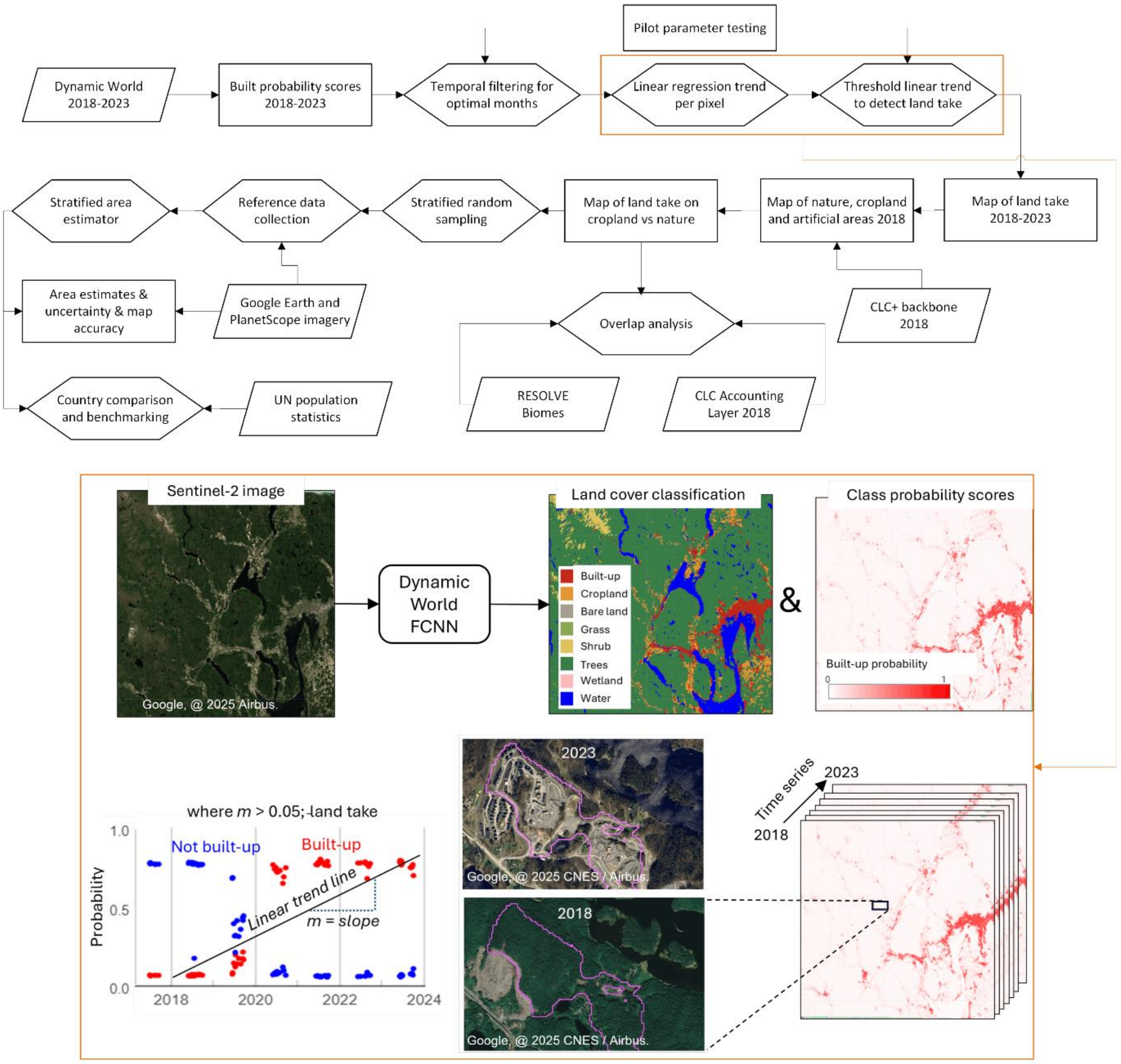


***Figure S1. Methodological workflow used to derive area estimates of land take.*** *The linear regression trend thresholding of built-up probability scores to detect land take is illustrated in more detail below the flow chart. The Dynamic World model processes Sentinel-2 imagery to generate land cover classifications with per-pixel probability scores for each class. To detect land take, we apply a linear regression to the time series of built-up class probabilities from 2018 to 2023. Pixels with a regression slope exceeding 0.05 are classified as experiencing land take. Aerial photographs: Google, @ 2025 Airbus. Abbreviations: CLC - Corine Land Cover, UN - United Nations.*

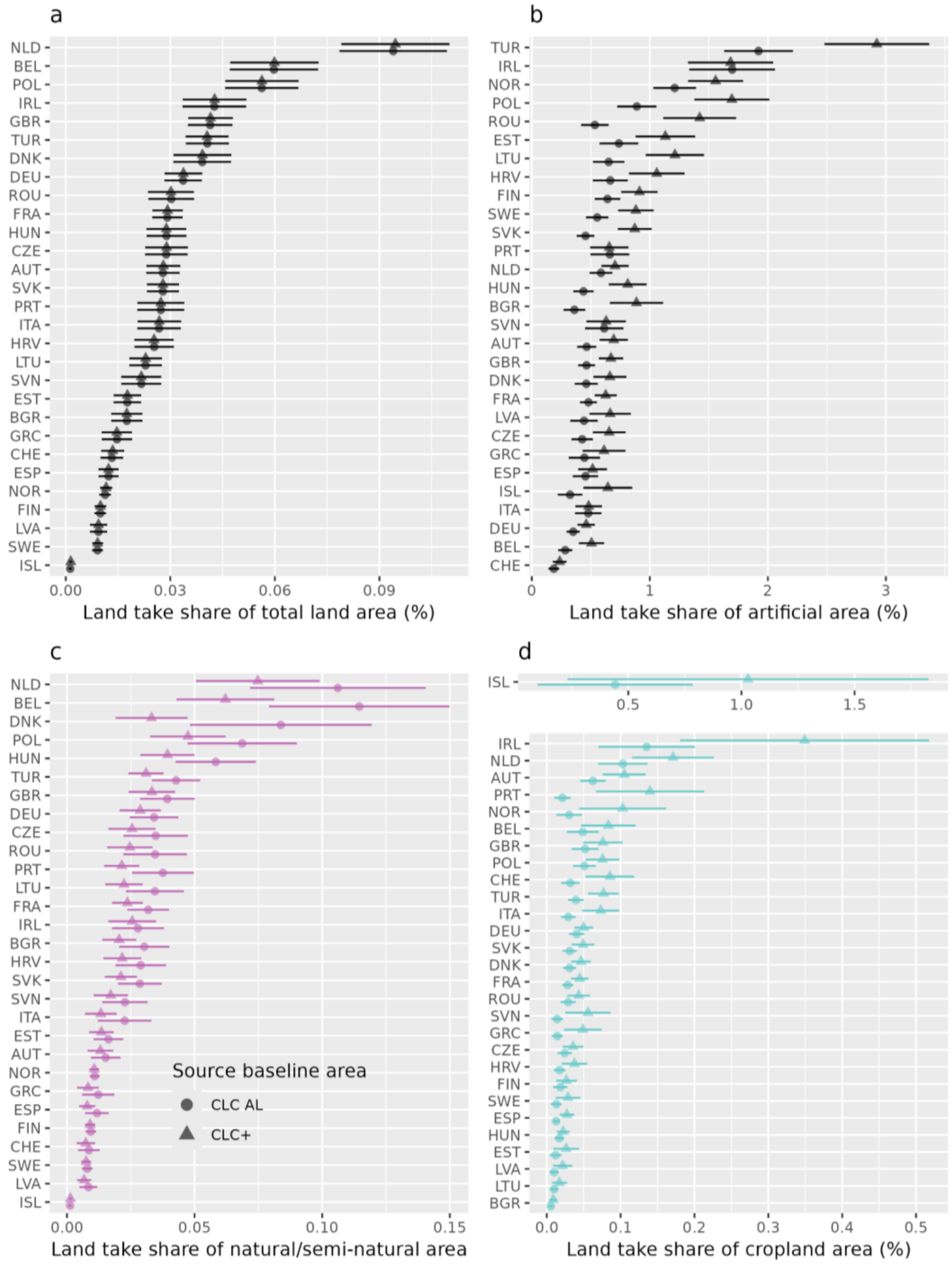


***Figure S2. Land take shares of total and baseline land cover area.*** *Land take expressed as a percentage of baseline terrestrial land area (a), and baseline artificial land cover area (b). Land take in natural/semi-natural land as a percentage of baseline natural land cover area (c). Land take of cropland as a percentage of baseline cropland land cover area (d). Unbiased area estimates are shown with points and 95% confidence intervals are shown with error bars. Confidence intervals are estimated from a sample size of n = 9893. An estimate based on baseline land cover area derived from Corine Land Cover Accounting Layer (CLC AL), and CLC backbone (CLC+) are displayed separately.*

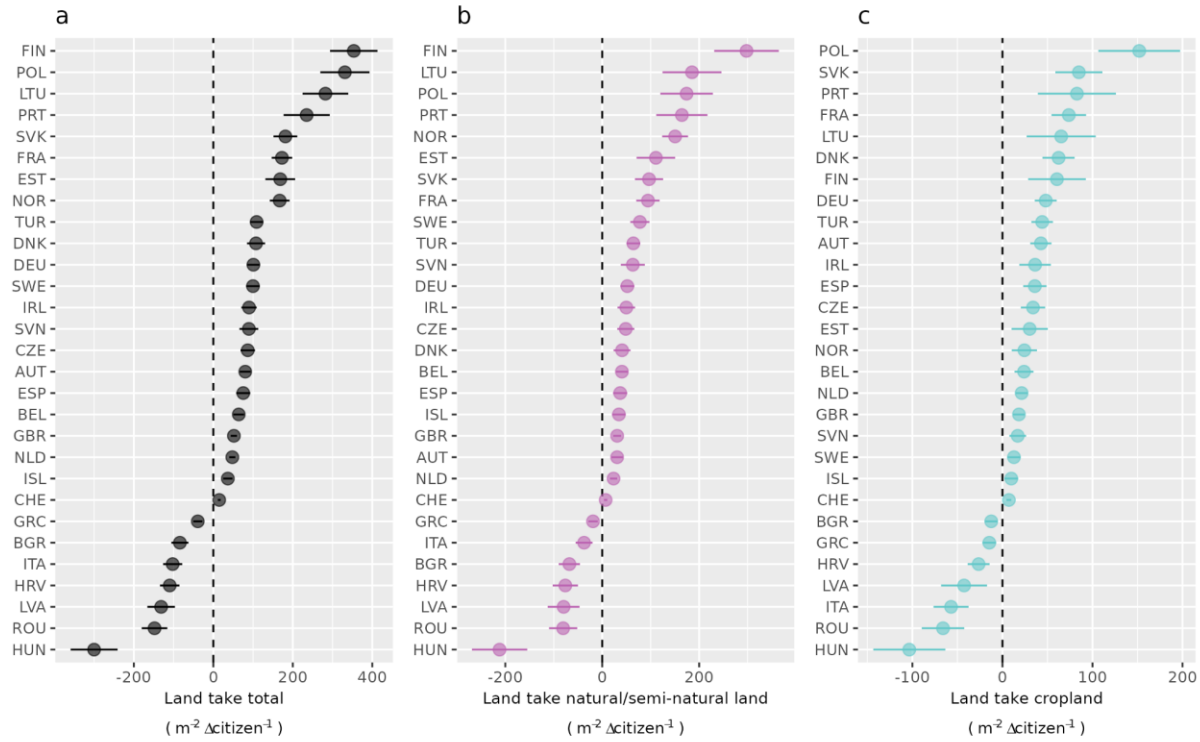


***Figure S3. Marginal land consumption, expressed as land take per unit of population change between 2018 and 2023****. a Marginal land consumption on all land. b Marginal land consumption on natural/semi-natural land. c Marginal land consumption on cropland. Design-based area estimates of land take, along with 95% confidence intervals, are divided by the corresponding population change over the same period.*

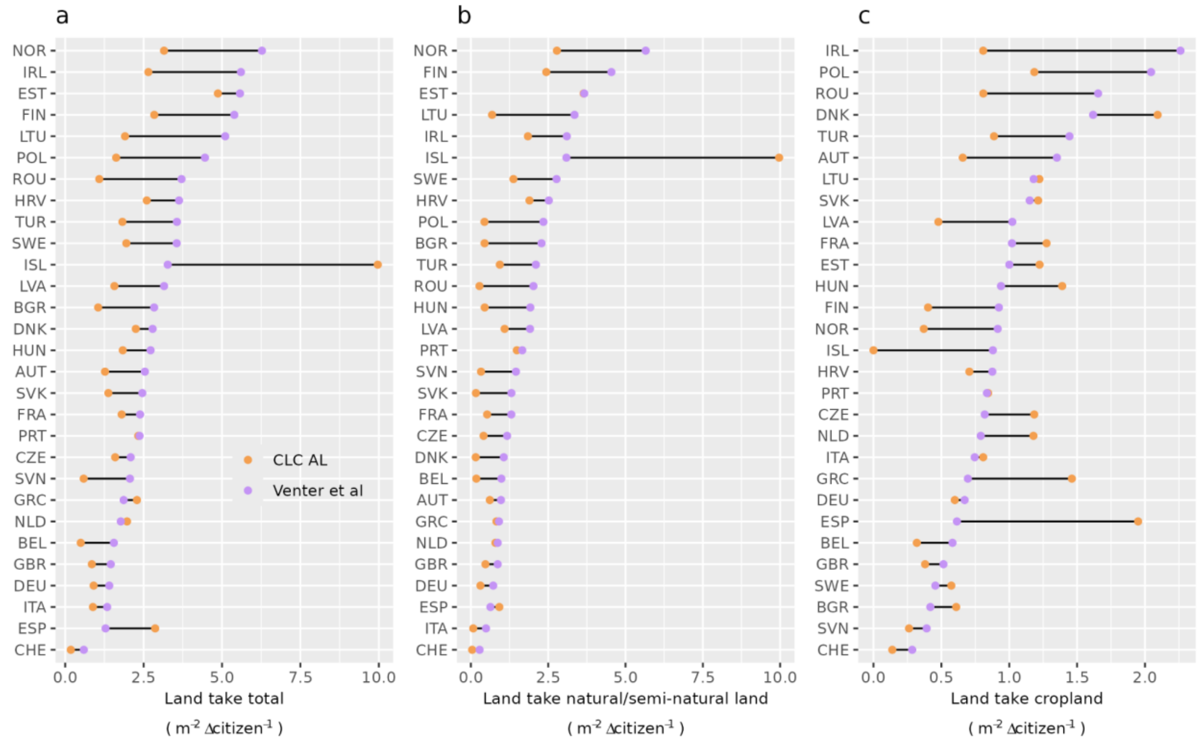


***Figure S4. Comparison in annual land take rates between those derived in our paper (2018-2023) and those derived from the Corine Land Cover Accounting Layer (2000-2018).*** *a Land take rates for all land.b Land take on natural/semi-natural land. c Land take on cropland.*

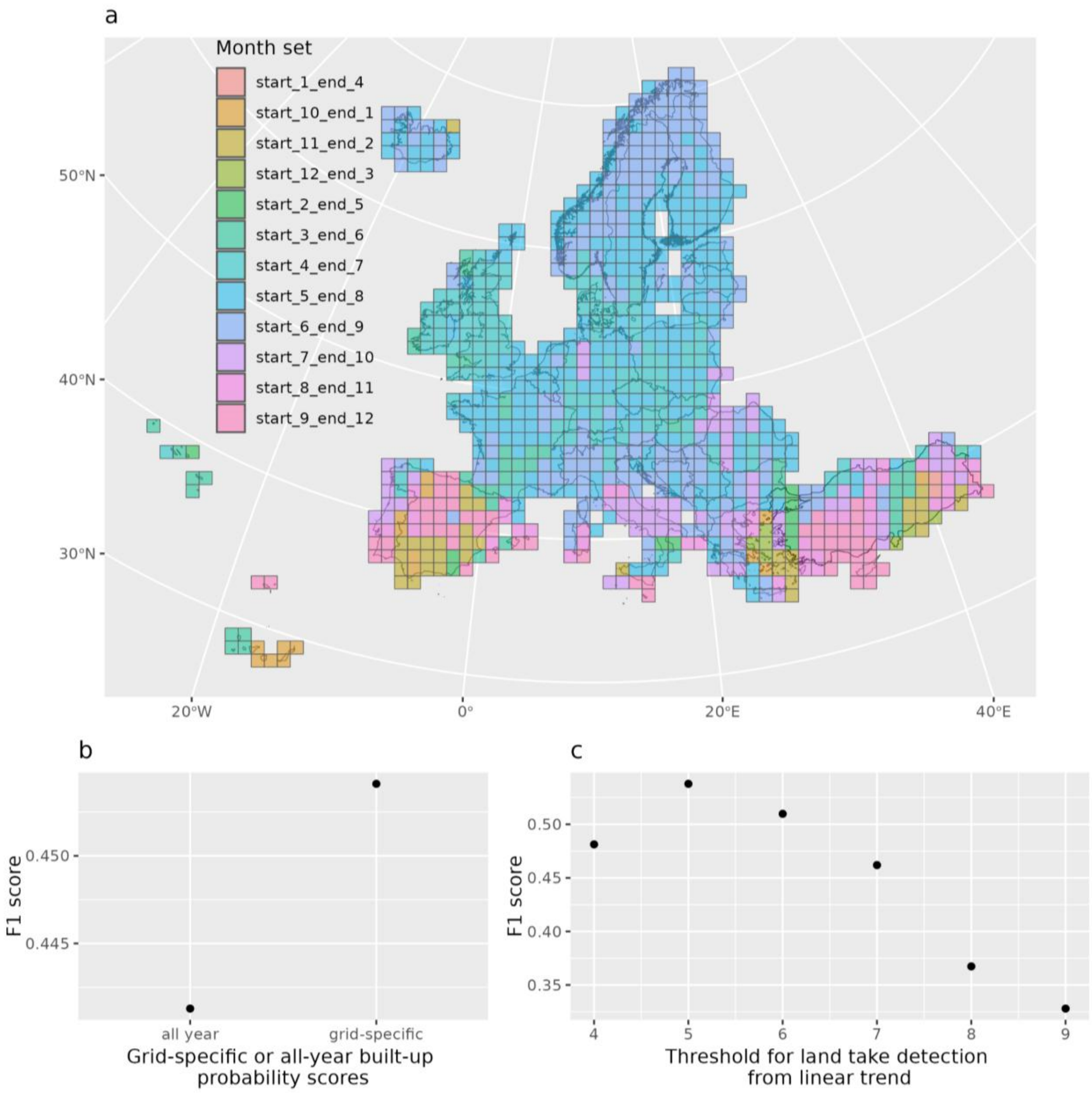


***Figure S5. Pilot testing of land take mapping algorithm parameters.*** *a Map of grid-specific months used for filtering Dynamic World built probability scores. b Results from testing whether to use all months in the year, or grid-specific months. c Results from testing the linear trend threshold for defining built-up expansion. Eurostat GISCO geospatial data used for country boundaries licensed under a Creative Commons Attribution 4.0 International License (https://creativecommons.org/licenses/by/4.0/) © EuroGeographics for the administrative boundaries.*

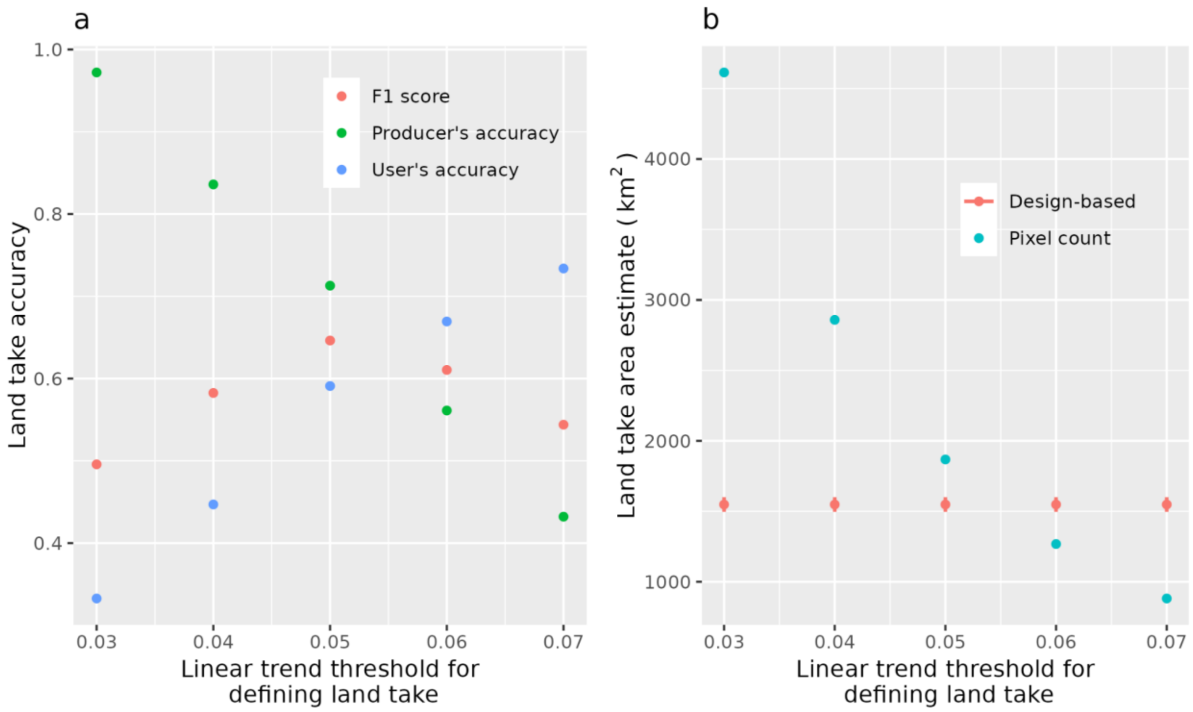


***Figure S6. Sensitivity analysis of linear trend threshold and buffer zone delineation.*** *a Accuracy of land take classification expressed across a range of linear trend thresholds. b Area estimates based on pixel counting and design-based methods across a range of linear trend thresholds. Error bars show 95% confidence intervals for the design-based estimates.*
