# Supplementary material for: Europe’s land take and the loss of nature and cropland to artificial surfaces
Source: Nat Commun. 2026 Apr 13;17:5122. doi: 10.1038/s41467-026-71931-w (PMC13247041; doi:10.1038/s41467-026-71931-w)
Supplement: Supplementary file 3 — Transparent Peer Review file [file 41467_2026_71931_MOESM3_ESM.pdf]

nature portfolio

Peer Review File

Europe’s land take and the loss of nature and cropland to artificial surfaces

Corresponding Author: Dr Zander Venter

Parts of this Peer Review File have been redacted as indicated to remove third-party material.

This file contains all reviewer reports in order by version, followed by all author rebuttals in order by version.

Version 0:

Reviewer comments:

Reviewer #1

(Remarks to the Author)
This is an important and timely piece of work, highly relevant to research on land take in Europe. It addresses a significant political need, which is to effectively monitor the dynamics of land take. It is clear that the classically used datasets have weaknesses—either they lack comprehensive territorial coverage when precise (e.g., Urban Atlas) or they lack fine spatial resolution (e.g., CLC).
This article aims to improve our understanding of these dynamics by using the Dynamic World dataset, which is regularly updated and offers very high spatial resolution. This dataset was combined with the CLC+ dataset to produce a mask identifying cropland and natural areas. A linear trend analysis was then conducted to determine probabilities of state change due to urbanization, and the entire process was manually controlled and validated by a team of interpreters. This is a substantial effort, invaluable both for advancing knowledge on land take dynamics and for policymakers, as it highlights the gap between the European goal of no net land take and recent trends in land consumption.

The methodology is robust, thoroughly detailed, and yields results of significant importance for the field of study. Yet, I must emphasize that I am not a remote sensing expert, and my ability to fully appreciate the scale of the work undertaken is limited. However, as an occasional user of some of the mentioned datasets, I fully agree with the identified research gaps and find the quality of the provided data entirely commensurate with the stakes involved.

Despite the importance of this work, I would like to suggest a few improvements to further enhance the document, which deserves publication.
Recommendations
First, regarding the article’s structure, I find it somewhat confusing. The methodology section appears after the discussion and conclusion. While I understand that the methodological details are complex and may be overwhelming for non-expert readers, it might have been better to move the bulk of this section to an appendix, retaining a concise methodological overview in the main text to improve readability.
The extensive focus on methodology seems to come at the expense of the discussion and conclusion, which feel somewhat underdeveloped. For instance, stating that land take targets are optimistic is not new—this has been widely discussed for years. Instead, it would have been interesting to introduce nuances in the definition of land take which has different implications depending on whether it involves light or heavy development. On average, only about 50% of land take areas are sealed, but this rate varies significantly by region.
Additionally, some conclusions seem to lack sufficient substantiation. For example, the claim that Switzerland has managed land development better than Norway—despite comparable economic development—due to its effective land use policy feels premature. Could this not be attributed to population density, land price, or simply scarcity in land availability?
More broadly, I would avoid using the term drivers to describe land use change. Drivers are better understood as the underlying determinants of land take (e.g., population growth, de-cohabitation, industrial development), not the changes themselves (p. 8).
Minor details:
• The acronym CLC AL is not defined at its first mention but only later.
• Denmark is not in Central Europe but in Northern Europe.
• I would replace, in the conclusion, the phrase "The dearth of information on fine-scale land take in Europe" by "The dearth of information on small-scale land use covering the whole of Europe," since data does exist—just not for the entire European territory.

(Remarks on code availability)

Reviewer #2

(Remarks to the Author)
In their paper "Europe's land take: revealing loss of nature and cropland to artificial surfaces", the authors produced high resolution land cover maps and analyzed land take in detail for European countries. I consider the paper a valuable contribution, because meeting ambitious policy goals also requires rigorous accounting at the right level of detail. There have been some papers that show and discus the loss of pervious surface at an even finer scale, something that you could also discuss:
Pauleit et al., 2005 10.1016/j.landurbplan.2004.03.009
Perry and Nawaz, 2008 10.1016/j.landurbplan.2007.12.004
Verbeeck et al.,2011 10.1016/j.landurbplan.2010.09.007
Warhurst et al., 2014 10.1016/j.scitotenv.2014.03.035
Lee et al., 2017 10.1016/j.ufug.2017.03.004
Strohbach et al., 2019 10.3389/fenvs.2019.00029
Hence, densification not only happens in the urban centers as you state in line 219, but also in the suburban and ex-urban areas.

I have major objection that I would like to be corrected: the use of the term "nature" and "ecosystem". Throughout the paper, the authors use "nature" to refer to all land cover types that are not developed and not cropland. This is really confusing, since a spruce forest or a highly productive grassland is as "unnatural" as cropland. This might lead us into philosophical terrain, but I would really suggest you refrain from using "nature". Your use of the term also leads to really awkward constructs, such as "stable nature". I would suggest using something like "other non-developed" or "natural and semi-natural land". Maybe you know of a better term, but you should not use "nature". Likewise, what you call "ecosystem types" in figure 3, are really land cover types.

As a minor note, I would suggest softening your statement in line 277. I think the goals are quite sincere. I think it is natural for the Commission to measure progress with existing data sets, since they are already being payed for by European tax payers. Nevertheless, you are absolutely right to suggest using something more sophisticated that also allows for detecting drivers. Nevertheless, I don't think the goals of the EU and it's institutions should be called into question.

Finally, your method produces interesting and high-resolution results. How do you consider it's repeatability in the future? You yourself mention that you had to leave out certain countries, because it took so much time.

(Remarks on code availability)
I would need extra time for checking the code, since I have no experience with earth engine.

Reviewer #3

(Remarks to the Author)
The study provides a high-resolution, design-based assessment of land take across Europe between 2018 and 2023, combining Sentinel-2 imagery, the Dynamic World land cover product, and probability-based area estimation to correct for classification bias. The authors find that Europe lost ~1543 ± 57 km² of natural or agricultural land per year, approximately 1.5–2× higher than previous European Environment Agency (EEA) estimates based on CORINE Land Cover (CLC) data.
This work represents a major methodological and empirical advance in monitoring European land take. It substantially improves spatial and temporal resolution over existing EEA products (10 m vs. 100–500 m) and provides the first design-based continental estimates aligned with ecosystem-accounting principles. The implications for EU policies—especially the no-net-land-take by 2050 target—are significant, as the results suggest that official statistics may underestimate true land consumption.
The manuscript's conclusions are well supported by the presented data and analytical approach. The use of design-based inference following Olofsson et al. (2014) represents best practice for producing unbiased area estimates from imperfect maps. Overall, the approach is statistically robust and transparently reported, and the uncertainty propagation is appropriate for a continental-scale study. The uncertainty quantification (95% CI) is correctly derived from stratified variance estimators. The authors also appropriately acknowledge the limitations—particularly the omission of net land take (i.e., restoration or de-artificialization processes).

Suggested Improvements
1. Statistical validation of the 0.05 threshold: the authors could strengthen the methodological transparency by providing a sensitivity or stability analysis showing how omission and commission errors vary across alternative slope thresholds (e.g., 0.03–0.07). This would demonstrate the robustness of the chosen 0.05 cut-off and help readers understand its effect on the final land-take estimates.
2. Quantitative assessment of buffer strata: Demonstrate (perhaps with simulation or subset validation) that the 0.03–0.05 range effectively captures likely omissions.

3. Discussion of Dynamic World limitations: Clarify potential regional biases (e.g., high-latitude snow cover, construction noise) and whether the seasonal filtering fully mitigates them.
4. Visual clarity: A schematic explaining how the trend regression and threshold translate into class transitions would aid comprehension for non-remote-sensing readers.

Minor comments:
Line 45-47: The authors citation of Chakraborty, T. et al. (2024) should be moved at the end of the line 47 sentence as the study it actually includes 10m resolution datasets.

(Remarks on code availability)

Version 1:

Reviewer comments:

Reviewer #1

(Remarks to the Author)
All the comments I raised during the first round of review have been addressed in a fully satisfactory manner. I also believe that the remarks made by the other reviewers (and the authors' responses to them) have further contributed to significantly improving the manuscript. As a result, this revised version now appears to be very well developed and, in my view, deserves to be published almost as is.
I nevertheless have two very minor comments:
• L2: please rephrase "human land use change" as "land use change", since land use is inherently a human-centered concept, which distinguishes it from the concept of land cover.
• L205: regarding the soil monitoring law, please replace "adopted recently" by "adopted by the European Parliament on 23 October 2025".
Thank you.

(Remarks on code availability)

Reviewer #2

(Remarks to the Author)
Thank you for thoroughly revising the paper. I look forward to seeing it published.

(Remarks on code availability)
I have tried to review the code, but it is hard without access to "ee.FeatureCollection('projects/nina/Arena/export_grid_EEA39')" In general, the code looks good and well documented.

Reviewer #3

(Remarks to the Author)
The authors have addressed all my comments and substantially improved the manuscript.

(Remarks on code availability)

Open Access This Peer Review File is licensed under a Creative Commons Attribution 4.0 International License, which permits use, sharing, adaptation, distribution and reproduction in any medium or format, as long as you give appropriate credit to the original author(s) and the source, provide a link to the Creative Commons license, and indicate if changes were made.
In cases where reviewers are anonymous, credit should be given to 'Anonymous Referee' and the source.
The images or other third party material in this Peer Review File are included in the article's Creative Commons license, unless indicated otherwise in a credit line to the material. If material is not included in the article's Creative Commons license and your intended use is not permitted by statutory regulation or exceeds the permitted use, you will need to obtain permission directly from the copyright holder.
To view a copy of this license, visit https://creativecommons.org/licenses/by/4.0/

We thank the reviewers for their constructive critiques of our work. The manuscript has been substantially revised to address all the reviewer comments with some additional analyses presented in the supplementary material. Our responses are in red text with quotes from the revised manuscript in blue text.

Reviewer #1 (Remarks to the Author):

This is an important and timely piece of work, highly relevant to research on land take in Europe. It addresses a significant political need, which is to effectively monitor the dynamics of land take. It is clear that the classically used datasets have weaknesses—either they lack comprehensive territorial coverage when precise (e.g., Urban Atlas) or they lack fine spatial resolution (e.g., CLC).

This article aims to improve our understanding of these dynamics by using the Dynamic World dataset, which is regularly updated and offers very high spatial resolution. This dataset was combined with the CLC+ dataset to produce a mask identifying cropland and natural areas. A linear trend analysis was then conducted to determine probabilities of state change due to urbanization, and the entire process was manually controlled and validated by a team of interpreters. This is a substantial effort, invaluable both for advancing knowledge on land take dynamics and for policymakers, as it highlights the gap between the European goal of no net land take and recent trends in land consumption.

The methodology is robust, thoroughly detailed, and yields results of significant importance for the field of study. Yet, I must emphasize that I am not a remote sensing expert, and my ability to fully appreciate the scale of the work undertaken is limited. However, as an occasional user of some of the mentioned datasets, I fully agree with the identified research gaps and find the quality of the provided data entirely commensurate with the stakes involved.

We appreciate the positive feedback.

Despite the importance of this work, I would like to suggest a few improvements to further enhance the document, which deserves publication.

Recommendations

First, regarding the article’s structure, I find it somewhat confusing. The methodology section appears after the discussion and conclusion. While I understand that the methodological details are complex and may be overwhelming for non-expert readers, it might have been better to move the bulk of this section to an appendix, retaining a concise methodological overview in the main text to improve readability.

We appreciate that the methods can be very detailed and not accessible for non-expert readers. To address this we have gone through and made changes or elaborated on potentially confusing sentences in the revised manuscript. In addition, we have expanded the methodology

figure (Supplementary Fig. 1) to include a schematic illustrating the core component of our land take mapping method in an attempt to make it more understandable by readers who are not remote sensing experts.

Regarding the structure and length: We have followed the Nature Communications editorial guidelines which require that the methods section is placed after the discussion and conclusion. The author guidelines also state “Methods are typically less than 3000 words.” and our text is currently (with revisions) at 2700 words. Therefore we do not think it is necessary to move the bulk of the methods to the appendix.

The extensive focus on methodology seems to come at the expense of the discussion and conclusion, which feel somewhat underdeveloped. For instance, stating that land take targets are optimistic is not new—this has been widely discussed for years. Instead, it would have been interesting to introduce nuances in the definition of land take which has different implications depending on whether it involves light or heavy development. On average, only about 50% of land take areas are sealed, but this rate varies significantly by region.

Thank you for this suggestion. In the revised version we have indeed tried to unpack the definition of land take a bit better, distinguishing soil sealing (permanently occupied by artificial surfaces) more clearly as a component of land take, and exploring what this distinction would mean quantitatively. (Yes, it seems that less than 50% of the taken areas are permanently sealed, although the permanent loss of vegetation is definitely higher.) And we have now also added a call for further comparative studies and standardisation of the different land-take-related concepts, for which we think that our methodology provides an excellent starting point.

The introduction now includes a more nuanced definition which acknowledges soil sealing:

“Artificialization, or in simpler words, land take is an important driver and component of habitat loss. In this process natural or semi-natural ecosystems (e.g. forests, grasslands, wetlands, etc.; henceforward “natural and semi-natural land”), and agroecosystems (actively managed arable and permanent crops; henceforward “cropland”) get destroyed for construction purposes and are entirely replaced with artificial ecosystems (i.e., built-up areas) 8,9. While some of the area taken may be rehabilitated or recolonised later, significant portions of the eradicated vegetation will be permanently covered in non-permeable artificial materials (also known as soil sealing), resulting in a permanent loss of ecological functionality.”

In the discussion we also benchmark our estimates against recent estimates for soil sealing:

“We found that Europe experienced an average of 1548 ± 27 km2 of land take annually between 2018 and 2023. In contrast, the EEA reports a gross land take of approximately 1000 km2 yr-1 between 2000 and 2018 (998 km2 yr-1 from the EEA statistical dashboard 17 and 1160 km2 yr-1 in a recent scientific publication 6). Although the aforementioned statistics are based on pixel counting, the EEA has done important work estimating the area of soil sealing using design-based methods, and report a rate of 413 km2 yr-1 between 2015 and 2018 18.

Soil sealing is one component of land take and therefore the estimates are not directly comparable with ours, however, our estimates are at least one and a half times larger than EEA land take estimates relying on coarse-resolution CLC Accounting Layers (Supplementary Fig. 4)."

And we emphasize how nuances in definitions can lead to different area estimates, but that our method allows for adjusting the target definition in the design-bases area estimation approach:

"This combination of methods is highly flexible: the final estimator pertains to the definition of land take applied in the reference dataset and can deviate from the definition used to create the map strata 19,45. Therefore, it can be flexibly applied to any consistent and meaningful, but objectively interpretable definition of land take, or related processes such as soil sealing, artificialization, etc. As comparing our results with similar other studies show, the details of the definition of land take (what is included and what is not) have a substantial influence on the final quantitative estimates, with permanently sealed surfaces only comprising up to ~30-40% of gross land take over Europe 18. Our methodology offers relatively straightforward pathways for creating and comparing quantitative estimations for different operative definitions of land take in various geographical contexts. This could be leveraged for improving the clarity and standardisation of key land take concepts in the future."

Additionally, some conclusions seem to lack sufficient substantiation. For example, the claim that Switzerland has managed land development better than Norway—despite comparable economic development—due to its effective land use policy feels premature. Could this not be attributed to population density, land price, or simply scarcity in land availability?

We agree that the speculation about what underlies differences in land take between Norway and Switzerland is premature and over-simplified. We have therefore altered the paragraph in the discussion to be more acknowledging of the complex drivers of land take and rather emphasize the need for more research on such differences between countries. The text now reads:

"Despite similar population growth rates to Norway, Switzerland exhibits the lowest rates of per capita land take. Switzerland's approach to land use is characterized by a collaborative effort between federal, cantonal, and municipal authorities, ensuring that land development aligns with national sustainability goals. Switzerland has shown relatively high compliance with strategic limits on the total area of building zones, as demonstrated in the canton of Zurich over a 20-year period [30]. However, attributing land use efficiency solely to policy is likely premature. Factors such as land scarcity, high land prices, and population density also strongly influence development patterns and the spatial footprint of urban growth [20,31]. Switzerland, for example, has among the highest population densities and topographical constraints in Europe, which may inherently limit expansive development. Further research is needed to quantify the relative contribution of planning frameworks versus structural drivers such as land availability and settlement preferences."

More broadly, I would avoid using the term drivers to describe land use change. Drivers are better understood as the underlying determinants of land take (e.g., population growth, de-cohabitation, industrial development), not the changes themselves (p. 8).

We agree that we have used the word “driver” too loosely without specifying its definition better. In the introduction we now qualify the “driver” concept clearly and thereafter ensure to replace all instances of the word “driver” with “land-use driver”. In the discussion we re-emphasize that these are proximate drivers and that we do not claim to conclude anything about the underlying drivers or determinants.

“Drivers can be classified as proximate and act locally, for example residential development, but often have distal causes that act regionally, for example national housing policies [20, 222]. Linking land take to industry- and land-use-specific proximal drivers may be particularly helpful in Europe where EU-level trade and industry policy can have significant impact on sustainable development targets.”

To elaborate a bit on our decision: we would argue that “driver” can be used in this context given that behind each land take type (ie. residential, transport etc.) there is a responsible industry that is driving the land use change. Of course these are not underlying determinants, but they are proximate drivers. Below we paste a passage from a much cited paper from Plieninger et al., (2016) which provides some context for our choice of language:

“The understanding of the reasons behind landscape changes has been at the center of recent landscape research, and the “driving forces”, i.e. the forces that drive changes in and of a landscape (Bürgi et al., 2004), have developed into a fundamental concept. Initially introduced in the late1990s as an indicator framework for environmental policy (OECD,1999), the concept of driving forces is now used as a framework for understanding the causes, processes, and outcomes of landscape change and has become indispensable for the evaluation of policy interventions (Klijn, 2004)....The concept of driving forces distinguishes between proximate and underlying drivers of change. ***Proximate drivers refer to human activities at the local level that result in landscape change, such as agricultural expansion or extension of settlements (Geistand Lambin, 2002)****. Underlying drivers comprise the fundamental social and natural processes (e.g. human population dynamics, agricultural policies, markets, or culturally embedded attitudes and beliefs) that underpin the proximate drivers and either operate at the local level or have a more indirect impact from the national or global level (Geist and Lambin, 2001; Geist and Lambin, 2002).”*

Plieninger, T., Draux, H., Fagerholm, N., Bieling, C., Bürgi, M., Kizos, T., Kuemmerle, T., Primdahl, J., & Verburg, P. H. (2016). The driving forces of landscape change in Europe: A systematic review of the evidence. Land Use Policy, 57, 204-214. https://doi.org/10.1016/j.landusepol.2016.04.040

Minor details:

• The acronym CLC AL is not defined at its first mention but only later.

This has now been corrected.

• Denmark is not in Central Europe but in Northern Europe.
We have replaced “Central Europe” with “Central-Northern Europe”.

• I would replace, in the conclusion, the phrase "The dearth of information on fine-scale land take in Europe" by "The dearth of information on small-scale land use covering the whole of Europe," since data does exist—just not for the entire European territory.
We have adjusted the entire sentence to both address your comment and another from Reviewer 2:

“The limited availability of accurate information on fine-scale land take covering Europe, and its land-use drivers, highlights a need to strengthen monitoring to track progress towards the EU’s ambition of achieving no net land take by 2050.”

Reviewer #2 (Remarks to the Author):

In their paper "Europe’s land take: revealing loss of nature and cropland to artificial surfaces", the authors produced high resolution land cover maps and analyzed land take in detail for European countries. I consider the paper a valuable contribution, because meeting ambitious policy goals also requires rigorous accounting at the right level of detail. There have been some papers that show and discus the loss of pervious surface at an even finer scale, something that you could also discuss:
Pauleit et al., 2005 10.1016/j.landurbplan.2004.03.009
Perry and Nawaz, 2008 10.1016/j.landurbplan.2007.12.004
Verbeeck et al.,2011 10.1016/j.landurbplan.2010.09.007
Warhurst et al., 2014 10.1016/j.scitotenv.2014.03.035
Lee et al., 2017 10.1016/j.ufug.2017.03.004
Strohbach et al., 2019 10.3389/fenvs.2019.00029
Hence, densification not only happens in the urban centers as you state in line 219, but also in the suburban and ex-urban areas.

We appreciate the suggestion to include these fine-scale studies on soil sealing and land take in residential areas. We have now added some of the references as a discussion point in the section “Priority areas for mitigation”:

“We found that 80% of land take occurs in artificial and cropland ecosystems, as defined by CLC. Urban greenspaces, often misclassified as already “taken” in coarse-resolution maps like CLC, are essential for human well-being, biodiversity, and climate resilience [33,34]. Protecting these areas aligns with the Nature Restoration Law and is particularly urgent given the increasing densification of urban centers. Infilling and densification also happens on a smaller scale, and the trend termed “hidden urbanization”, whereby domestic gardens and residential areas are increasingly sealed with artificial and impervious surfaces can have significant consequences for urban flooding [35–37].”

I have major objection that I would like to be corrected: the use of the term "nature" and "ecosystem". Throughout the paper, the authors use "nature" to refer to all land cover types that are not developed and not cropland. This is really confusing, since a spruce forest or a highly productive grassland is as "unnatural" as cropland. This might lead us into philosophical terrain, but I would really suggest you refrain from using "nature". Your use of the term also leads to really awkward constructs, such as "stable nature". I would suggest using something like "other non-developed" or "natural and semi-natural land". Maybe you know of a better term, but you should not use "nature". Likewise, what you call "ecosystem types" in figure 3, are really land cover types.

We agree that the use of “nature” leads to ambiguity and is imprecise. We have now followed your suggestion and replaced “nature” with “natural and semi-natural land” in the text, and due

to space constraints “natural/semi-natural” in the figures and tables. We have also added this paragraph defining croplands versus natural and semi-natural land in the introduction:

“People have shaped European land systems for millennia, creating a continuum from undisturbed natural areas to semi-natural landscapes and heavily transformed urban or industrial zones characterized by artificial surfaces 7. Artificialization, or in simpler words, land take is an important driver and component of habitat loss. In this process natural or semi-natural ecosystems (e.g. forests, grasslands, wetlands, etc.; henceforward “natural and semi-natural land”), and agroecosystems (actively managed arable and permanent crops; henceforward “cropland”) get destroyed for construction purposes and are entirely replaced with artificial ecosystems (i.e., built-up areas) 8,9.”

We would defend the use of “ecosystem types” in figure 3 because we have a direct cross-walk from CLC AL to MAES, and that we use “ecosystem” in the ecosystem accounting sense of the word

As a minor note, I would suggest softening your statement in line 277. I think the goals are quite sincere. I think it is natural for the Commission to measure progress with existing data sets, since they are already being payed for by European tax payers. Nevertheless, you are absolutely right to suggest using something more sophisticated that also allows for detecting drivers. Nevertheless, I don't think the goals of the EU and it's institutions should be called into question.
We agree completely and do not mean to question the sincerity of the goals. We have revised the sentence to avoid this interpretation:

“The limited availability of accurate information on fine-scale land take covering Europe, and its land-use drivers, highlights a need to strengthen monitoring to track progress towards the EU’s ambition of achieving no net land take by 2050.”

Finally, your method produces interesting and high-resolution results. How do you consider it's repeatability in the future? You yourself mention that you had to leave out certain countries, because it took so much time.

Thank you for raising this question. We have now included more details about the most labour-intensive and reproducibility-critical steps of the analysis: the generation of the reference dataset, as well as about the app that we built to support this work. We also added a paragraph in the discussion to elaborate on the effort it required to produce country-specific area estimates in our study. Without knowledge of the specific resources available at institutions like the European Environmental Agency, it is difficult for us to speculate about whether this is practically repeatable, or something that could be operationalized into an annual monitoring tool. However, we hope the extra details in the text we provided will give useful guidance for those wanting to reproduce it. All the code and data will be publicly available upon publication and we hope it can be used further.

Please refer to the highlighted changes in the discussion section titled “Future prospects for land take monitoring”. The changes are too numerous to copy and paste here.

Reviewer #3 (Remarks to the Author):

The study provides a high-resolution, design-based assessment of land take across Europe between 2018 and 2023, combining Sentinel-2 imagery, the Dynamic World land cover product, and probability-based area estimation to correct for classification bias. The authors find that Europe lost ~1543 ± 57 km² of natural or agricultural land per year, approximately 1.5–2× higher than previous European Environment Agency (EEA) estimates based on CORINE Land Cover (CLC) data. This work represents a major methodological and empirical advance in monitoring European land take. It substantially improves spatial and temporal resolution over existing EEA products (10 m vs. 100–500 m) and provides the first design-based continental estimates aligned with ecosystem-accounting principles. The implications for EU policies—especially the no-net-land-take by 2050 target—are significant, as the results suggest that official statistics may underestimate true land consumption.

The manuscript's conclusions are well supported by the presented data and analytical approach. The use of design-based inference following Olofsson et al. (2014) represents best practice for producing unbiased area estimates from imperfect maps. Overall, the approach is statistically robust and transparently reported, and the uncertainty propagation is appropriate for a continental-scale study. The uncertainty quantification (95% CI) is correctly derived from stratified variance estimators. The authors also appropriately acknowledge the limitations—particularly the omission of net land take (i.e., restoration or de-artificialization processes).

We appreciate the positive feedback.

Suggested Improvements

1. Statistical validation of the 0.05 threshold: the authors could strengthen the methodological transparency by providing a sensitivity or stability analysis showing how omission and commission errors vary across alternative slope thresholds (e.g., 0.03–0.07). This would demonstrate the robustness of the chosen 0.05 cut-off and help readers understand its effect on the final land-take estimates.

While our initial "calibration" sample of 1000 locations was used to identify optimal slope threshold, we completely agree that a sensitivity analysis of the final map is in order. We have followed your advice and tested the effect of slope threshold on land take map accuracy/error and on the derived area estimates. We include an additional section in the methods of the paper, supported by Supplementary Fig. 6. I paste the text here for you:

"***Validation and sensitivity analysis***

The design-based area estimation produced unbiased measures of map accuracy per stratum per country (Supplementary Table 4). In theory, area estimates from a stratified estimator are robust to map inaccuracies, as they are derived from the reference sample rather than from

mapped areas based on pixel counts [18]. However, map accuracy can influence the precision of the estimate, reflected in the width of the 95% confidence intervals.

In the initial sampling phase (1,000 locations), we calibrated our algorithm to detect land take with optimal accuracy, identifying a linear trend threshold of 0.05 for built-up probability scores. Specifically, we identified a linear trend threshold for built-up probability scores of 0.05. To test the robustness of this threshold we performed a post-hoc sensitivity analysis using the full reference sample (n = 9,893). We generated new maps using linear trend thresholds ranging from 0.03 to 0.07, extracted area weights and post-stratified the original reference sample. Stehman et al (2020) [48] provide a post-stratified area estimator where the strata differ from the map classes. This estimator is appropriate for our sensitivity analysis given that the new maps, generated with different trend thresholds, produce new map classes for the reference sample. The post-stratified estimator produced unbiased map accuracy metrics per trend threshold which supported the choice of a 0.05 threshold. The 0.05 threshold produced a land take map with the highest F1 score and therefore the best balance between omission and commission error (Supplementary Fig. 6a). While the pixel counting area estimates steadily declined with increasing linear trend threshold the design-based estimates remained stable (Supplementary Fig. 6b)."

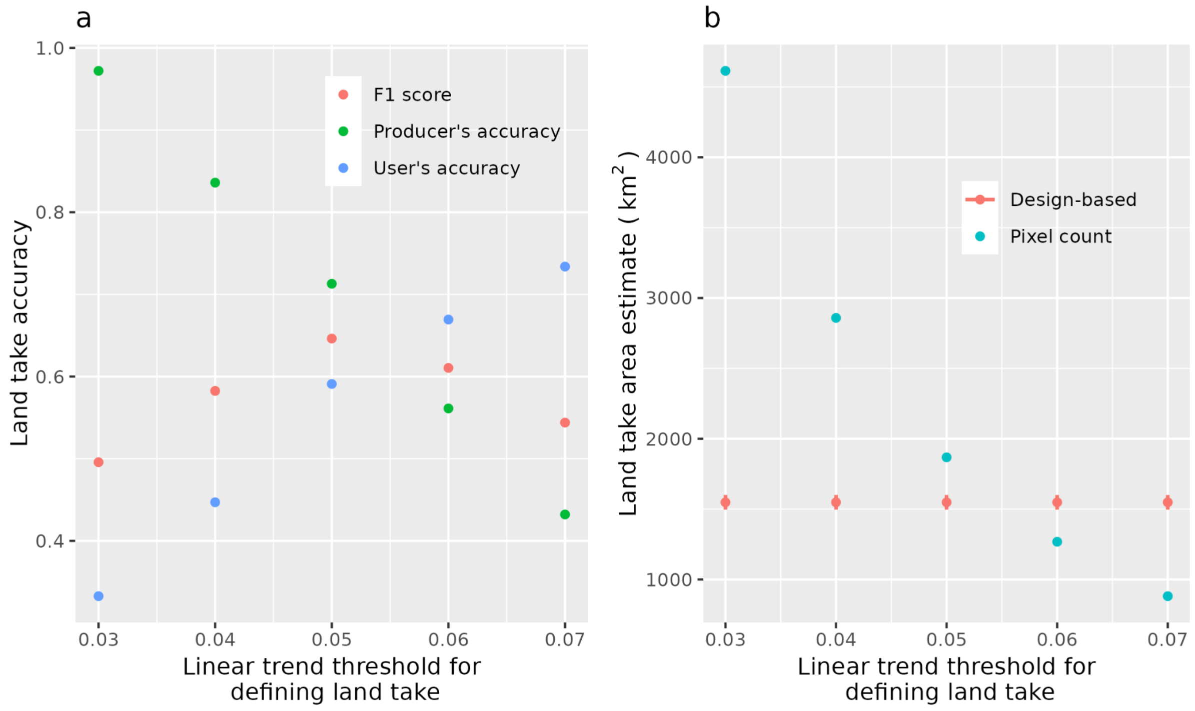

Figure S6. Sensitivity analysis of linear trend threshold and buffer zone delineation. a Accuracy of land take classification expressed across a range of linear trend thresholds. b Area estimates based on pixel counting and design-based methods across a range of linear trend thresholds. Error bars show 95% confidence intervals for the design-based estimates.

2. Quantitative assessment of buffer strata: Demonstrate (perhaps with simulation or subset validation) that the 0.03–0.05 range effectively captures likely omissions.

You raise a challenging and important point about buffer delineation in design-based area estimation. The seminal paper on mitigating the effects of omission errors in area estimation (Olofsson et al 2020) did not provide specific guidance on how to choose a buffer zone. Instead they called for more research on how to contain, mitigate and potentially eliminate the effects of omissions errors. Subsequent papers have used somewhat arbitrary buffer zones based on distances from the map class of interest (e.g. 90m from a deforestation pixel, Arevalo et al 2020), or, like in our analysis, based on probability scores (e.g. probability of fire disturbance, Reiche et al 2021). Therefore there is little empirical evidence to inform the choice of an optimal buffer size, and, like in previous papers, we were not able to perform a thorough sensitivity analysis due to the substantial effort it would require in collecting reference data for a range of buffer delineations. However, what remains clear from the foundational work by Olofsson et al (2020) is that the buffer size delineation and its ability to capture errors of omission will not bias the resulting area estimate of the focal map class (in our case; land take). Instead it will only affect the precision of the estimate. Therefore, even if we did not select the optimal buffer range, our area estimates may be less precise, but they would not be biased.

We have added the following text to the methods section to explain this to the reader:

"We defined the buffer stratum as pixels with intermediate trends in built-up probability scores of between 0.03 and 0.05. This buffer range was chosen based on visual inspection of the map where it was found that most land take omissions occurred within the 0.03 to 0.05 range. A thorough sensitivity analysis of varying buffer thresholds and widths would require a substantial amount of reference data collection, and empirical evidence for buffer selection guidelines are currently lacking [44]. Nevertheless, our inability to quantitatively prove the best buffer size (due to lack of stratified reference samples for each alternative buffer) does not invalidate the method. A poorly defined buffer will increase the standard error and widen the confidence interval because it reduces the likelihood of detecting omission errors, however it will not bias the area estimate itself [44,45]."

Arévalo, P., Olofsson, P. & Woodcock, C. E. Continuous monitoring of land change activities and post-disturbance dynamics from Landsat time series: A test methodology for REDD+ reporting. Remote Sensing of Environment 238, 111051 (2020).

Olofsson, P. et al. Mitigating the effects of omission errors on area and area change estimates. Remote Sensing of Environment 236, 111492 (2020).

Reiche, J. et al. Forest disturbance alerts for the Congo Basin using Sentinel-1. Environ. Res. Lett. 16, 024005 (2021).

3. Discussion of Dynamic World limitations: Clarify potential regional biases (e.g., high-latitude snow cover, construction noise) and whether the seasonal filtering fully mitigates them.

We have added a paragraph to the discussion to explicitly mention the challenge of map errors which may vary spatially due to climating or seasonal variation across Europe. As you will see in the text, we do not claim to fully mitigate these errors with seasonal filtering. However, the design-based estimation mitigates the risk of these biases being propagated to the final area estimates per country.

“A further advantage of design-based methods is that although map errors may vary regionally depending on, for example, cloud and snow cover contamination of satellite imagery, the final area estimates per country will remain unbiased. Despite our efforts, such as seasonal filtering (Supplementary Fig. 5), some regional variation in land take detection accuracy may persist. However, our design-based sampling strategy, which stratifies by both country and map class, effectively corrects for such spatial biases in the final area estimates. As a result, any regional inconsistencies would primarily increase the uncertainty (wider confidence intervals) around the estimates, but would not systematically bias the estimates themselves.”

4. Visual clarity: A schematic explaining how the trend regression and threshold translate into class transitions would aid comprehension for non-remote-sensing readers.

Supplementary Figure 1 has been updated to include a schematic of how the regression trend threshold translates into land take classification. We paste the schematic here for your convenience:

[Figure Redacted]

An excerpt from the figure caption: The Dynamic World model processes Sentinel-2 imagery to generate land cover classifications with per-pixel probability scores for each class. To detect land take, we apply a linear regression to the time series of built-up class probabilities from 2018 to 2023. Pixels with a regression slope exceeding 0.05 are classified as experiencing land take.

Minor comments:

Line 45-47: The authors citation of Chakraborty, T. et al. (2024) should be moved at the end of the line 47 sentence as the study it actually includes 10m resolution datasets.

We have done as you suggested.

Please find our responses in red below.

Reviewer #1 (Remarks to the Author):

All the comments I raised during the first round of review have been addressed in a fully satisfactory manner. I also believe that the remarks made by the other reviewers (and the authors' responses to them) have further contributed to significantly improving the manuscript. As a result, this revised version now appears to be very well developed and, in my view, deserves to be published almost as is.
I nevertheless have two very minor comments:
• L2: please rephrase "human land use change" as "land use change", since land use is inherently a human-centered concept, which distinguishes it from the concept of land cover.

We have excluded "human" from this phrase in the second line of the introduction.

• L205: regarding the soil monitoring law, please replace "adopted recently" by "adopted by the European Parliament on 23 October 2025".
Thank you.

We have added the phrase "adopted by the European Parliament on 23 October 2025" to this sentence.

Reviewer #2 (Remarks to the Author):

Thank you for thoroughly revising the paper. I look forward to seeing it published.

I have tried to review the code, but it is hard without access to "ee.FeatureCollection('projects/nina/Arena/export_grid_EEA39')" In general, the code looks good and well documented.
We have ensured that the Google Earth Engine assets including this ee.FeatureCollection are now publicly accessible.

Reviewer #3 (Remarks to the Author):

The authors have addressed all my comments and substantially improved the manuscript.
